# Chronic active non-lethal human-type tuberculosis in a high royal Bavarian officer of Napoleonic times–a mummy study

**DOI:** 10.1371/journal.pone.0249955

**Published:** 2021-05-04

**Authors:** Andreas G. Nerlich, Sonja M. Kirchhoff, Stephanie Panzer, Christine Lehn, Beatrice E. Bachmeier, Birgit Bayer, Katja Anslinger, Pascale Röcker, Oliver K. Peschel

**Affiliations:** 1 Institute of Pathology, Academic Clinic München-Bogenhausen, Munich, Germany; 2 Department of Radiology, Ludwig-Maximilians University (LMU), Munich, Germany; 3 Department of Radiology, Trauma Center Murnau, Murnau, Germany; 4 Paracelsus Medical University Salzburg, Salzburg, Austria; 5 Institute of Legal Medicine, Ludwig-Maximilians University (LMU), Munich, Germany; 6 Institute of Laboratory Medicine, University Hospital, Ludwig-Maximilians University (LMU), Munich, Germany; Hebrew University, ISRAEL

## Abstract

In paleopathology, morphological and molecular evidence for infection by mycobacteria of the *M*. *tuberculosis* complex (MTC) is frequently associated with early death. In the present report, we describe a multidisciplinary study of a well-preserved mummy from Napoleonic times with a long-standing tuberculous infection by *M*. *tuberculosis senso stricto* who died at the age of 88 years of focal and non-MTB related bronchopneumonia. The well-preserved natural mummy of the Royal Bavarian General, Count Heinrich LII Reuss-Köstritz (1763–1851 CE), was extensively investigated by macro- and histomorphology, whole body CT scans and organ radiography, various molecular tissue analyses, including stable isotope analysis and molecular genetic tests. We identified signs for a long-standing, but terminally inactive pulmonary tuberculosis, tuberculous destruction of the second lumbar vertebral body, and a large tuberculous abscess in the right (retroperitoneal) psoas region (a cold abscess). This cold abscess harboured an active tuberculous infection as evidenced by histological and molecular tests. Radiological and histological analysis further revealed extensive arteriosclerosis with (non-obliterating) coronary and significant carotid arteriosclerosis, healthy bone tissue without evidence of age-related osteopenia, evidence for diffuse idiopathic skeletal hyperostosis and mild osteoarthrosis of few joints. This suggests excellent living conditions correlating well with his diet indicated by stable isotope results and literary evidence. Despite the clear evidence of a tuberculous cold abscess with bacterioscopic and molecular proof for a persisting MTC infection of a human-type *M*. *tuberculosis* strain, we can exclude the chronic MTC infection as cause of death. The detection of MTC in historic individuals should therefore be interpreted with great caution and include further data, such as their nutritional status.

## Introduction

Human remains (mummies and skeletons) from previous cultures represent a highly valuable bioarchive suitable for the reconstruction of living and disease conditions in past populations, including evidence for infectious diseases, trauma and certain metabolic disorders [1]. Due to modern analytical techniques an increasing spectrum of information can successfully be retrieved. Likewise, the recent significant advances in radiological techniques, molecular tests (ancient DNA, “proteomics”), radiocarbon (absolute dating) as well as stable isotope analysis (diet, localisation of origin) provide a growing body of valuable information on historic individuals. In this regard, complete mummies are much more informative than mummy parts or skeletal remains alone [2]. Our group has recently shown the capability of such multidisciplinary mummy studies on an unknown Inca mummy that had originally been claimed to represent a bog body [3].

Particular attention has recently been paid to various infectious diseases, since these diseases have been assumed as to be major causes of death in past populations [4]. In this regard, particularly the successful molecular identification of ancient pathogen DNA (first accomplished in 1993 by Spigelman and Lemma [5]) opened the way for a better understanding of the role of infectious diseases. The first identified pathogen was the human-pathogenic *M*. *tuberculosis* complex (MTC); however, despite numerous studies gathering ample information about infection frequencies, and the appearance of various substrains involved in the dissemination of the disease in the last 5,000 years [6–9], still many questions remain open. Tuberculosis was one of the leading causes of death in 18^th^/19^th^ century Old World populations [10]. In consequence, in most instances the molecular proof of an infection by the MTC has been assumed to reduce the affected individual´s life expectancy. Also there exist explicit data showing an average survival period of untreated active tuberculosis cases of only 14 months [11]. In some reports the infection has even been directly linked with the cause of death [12,13] which is plausible in active, but not in latent disease [14]. Therefore, each case with a proven long-standing active disease, but without intimate link to the cause of death, is very instructive.

As described below more in detail, the Royal Bavarian General, Count Heinrich LII. Reuss-Köstritz, obviously suffered from a longstanding tuberculous infection with a chronically active cold abscess at the right psoas muscle, but died of a non-related disease, most obviously due to acute (broncho-)pneumonia. Furthermore, several data rule out any evidence of malnutrition, as can happen in chronic tuberculosis, or vitamin deficiency, and more importantly, indicate concurrent direct and indirect sequelae of arteriosclerosis. In summary, this historic individual provides clear evidence of a long-term case of active tuberculosis that was non-lethal and suggests that other such cases should be sought in order to understand the outcome of non-lethal active tuberculosis in history.

## Material and methods

### Count Heinrich LII. Reuss-Köstritz—The mummy

The well-preserved mummy of Count Heinrich LII. Reuss-Köstritz was removed from his burial place in November 2011 due to renovation works of the crypt where he had lain since his death in 1851. The permission for the removal from the crypt, and the subsequent extensive investigation, had been previously granted by the church authorities of the Bistum Regensburg (diocese) by Generalvikar Monsignore Fuchs, on behalf of the Ordinariatskonferenz´s resolution by the 5.10.2011.

The small aristocratic crypt had been erected in 1836 by Baron Friedrich Wilhelm von Jordan, a close friend of Count Heinrich, near the village of Dötting/ Wackerstein close to the river Danube, approximately 80 km north of Munich. A brief summary of Count Heinrich LII.’s life is presented in S1 File. For further information, the reader is referred to the extensive historical biography of Count Heinrich (in German) which covers not only the Count´s life, but also the complete family history of the members of this branch. This biography [15] also presents all the details of his activities and events; therefore, this is a supplement to the paleopathological investigation given in this paper.

**Fig 1:**
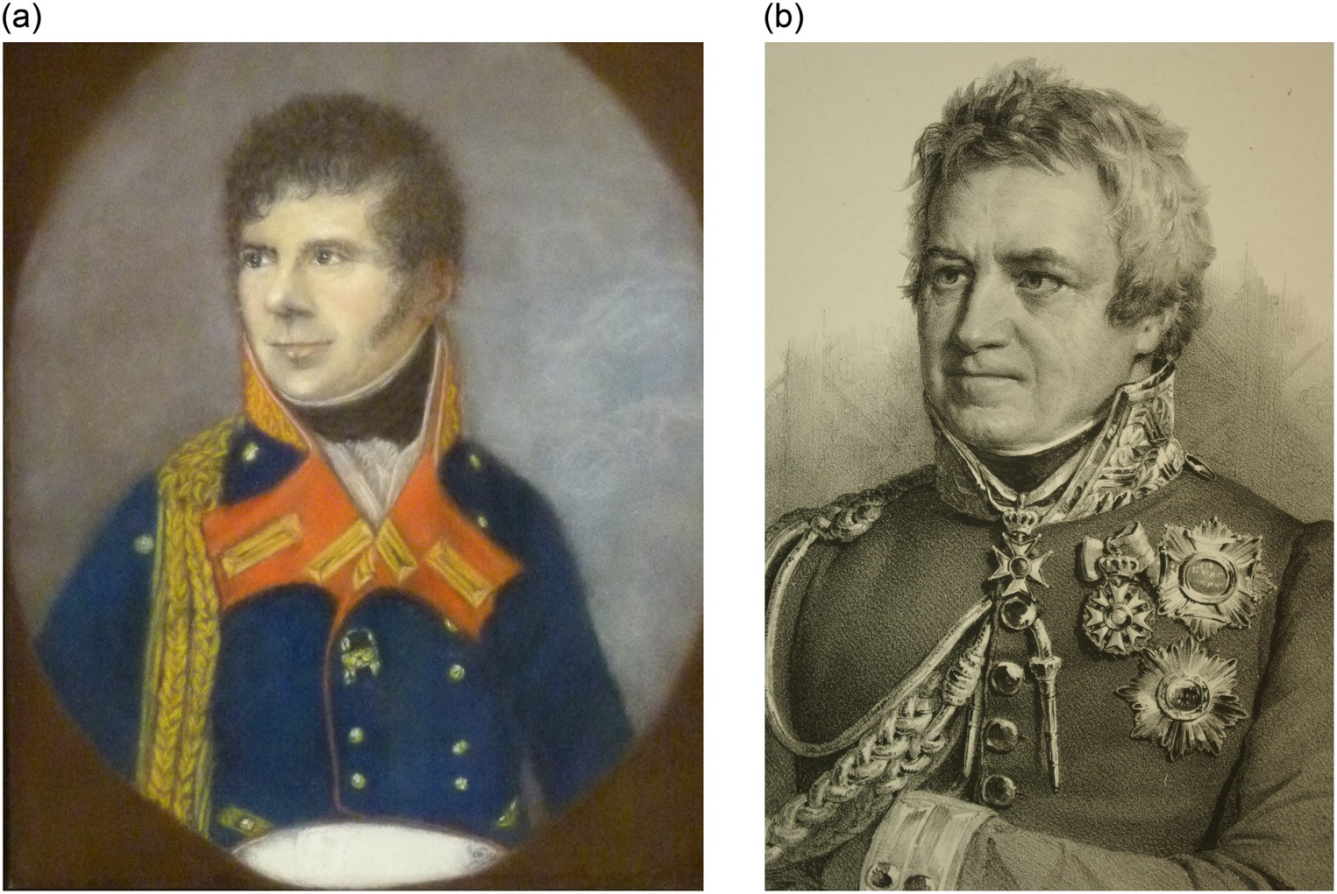
A: Count Heinrich LII Reuss-Köstritz as Royal Bavarian General Major and Adjutant of the Bavarian King, around 1806; oil portrait by Leberecht Vogel von Vogelstein [Museum und Kunstsammlung Schloss Hinterglauchau, Germany; with permission]. B. Count Heinrich LII Reuss-Köstritz around 1833 at the age of 70 years. Lithography by Hanfstaegel [Prof. Dr. A. Nerlich, München, with permission].

Briefly, Count Heinrich LII. Reuss-Köstritz (Fig 1) was born in 1763 CE as the third son of the youngest branch of the Köstritz family of the Thuringian sovereign family of Reuss younger line (Reuss jüngerer Linie). Despite his high princely status, for economic reasons, he was forced to look for an adequate position outside his tiny homeland. Accordingly, in 1780 CE, he entered the military service of the Electorate of Palatinate-Bavaria, where he was engaged, with promotions in military ranks, in several of the Napoleonic wars; the Second (1799–1801 CE), Third (1804–1805 CE), Fourth (1806–1807 CE) and Fifth Coalitions War (1809 CE); finally reaching the position, from 1806 CE onwards, of General Lieutenant and General Adjutant to the Elector (then King) of Bavaria, Maximilian I. Joseph. In the latter position, Count Heinrich was also frequently a travelling companion of the King and several of the royal family members. There is a report that Count Heinrich became seriously ill in 1822–23 CE, during a journey to Saxony, on the occasion of wedding preparations for one of the Bavarian king´s daughters. This disease episode, which unfortunately does not provide relevant medical data, was so severe that Count Heinrich was forced to interrupt his royal services for several months. From our present day view, it remains unclear whether this was related to the subsequent discovery of non-lethal tuberculosis or not [15]. After recovering, he resumed his adjutancy services in Bavaria and, despite an official demission from his post in 1825, he stayed close to the Bavarian Royal House until his death in 1851 at the age of 88 years. Even during the last years of his life he was very active, travelled a lot, and still carried out duties for the Bavarian Protestant church. Although we have only limited first and second hand information on his life, there is no evidence for any further (longer) absence from his services and duties up to his death [15]. Similarly, none of the reports indicate any further significant disease history.

After death, his body lay in repose in Munich for three days and was then transferred to the aforementioned crypt. According to written sources, the corpse remained without any conservation treatment so that we can assume the particular climatic conditions of the crypt building to be responsible for the natural mummification and good preservation of the body.

**Fig 2:**
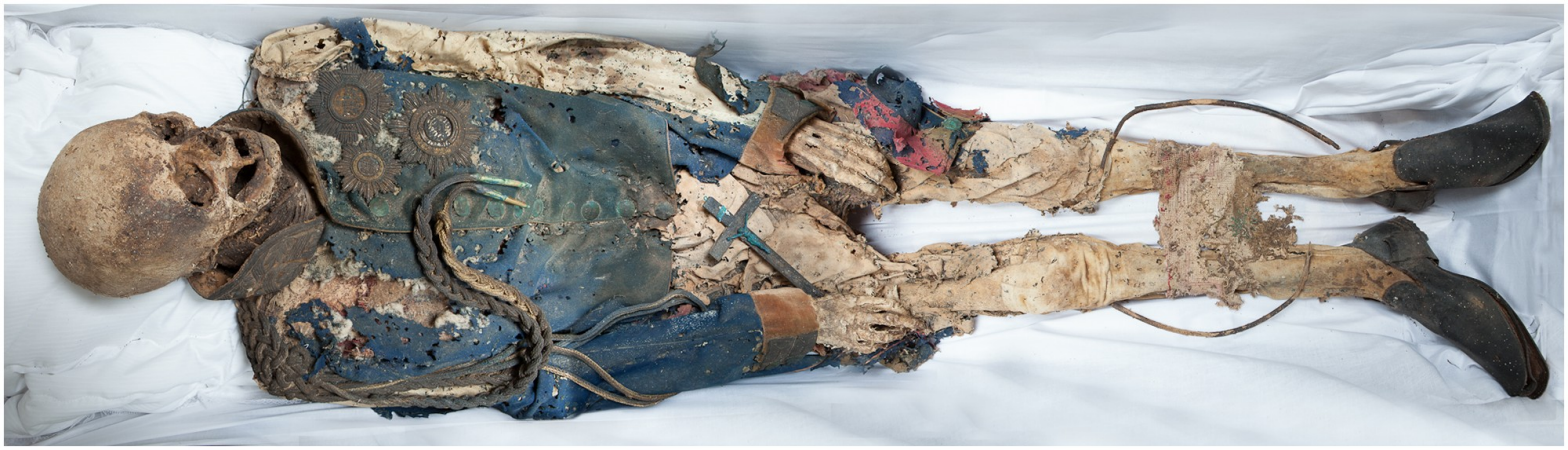
The mummy of General Count Heinrich LII. Reuss-Köstritz in his sarcophagus (after finishing the investigation).

**Fig 3:**
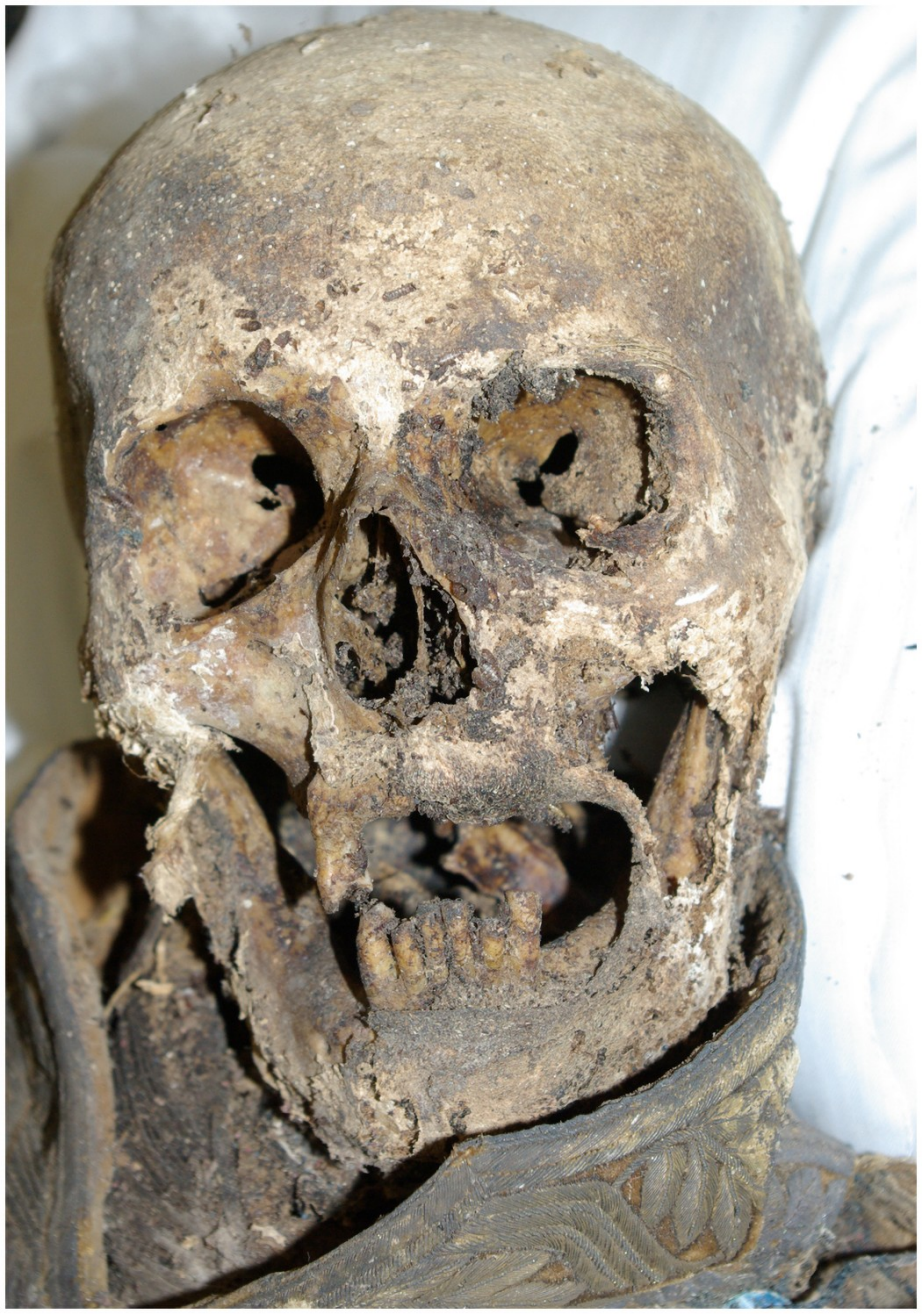
Skull of the mummy during the investigation. Here, particularly, the dental status can be evaluated.

After having opened the sarcophagus, we found the body of Count Heinrich LII. Reuss-Köstritz in his general´s uniform with the typical insignia of a royal General Adjutant (Fig 2). The external and internal soft tissue of the skull was significantly decomposed, allowing easy access to the interior of the skull through the foramen magnum where we found only small remnants of overt brain tissue. The status of the dentition was also easily determined by external inspection (Fig 3). The remainder of the body was tightly enveloped in his uniform so we decided first to perform a whole-body computed tomography (CT) scan. All direct examinations of the mummy took place in the summer of 2012.

### Paleoradiological CT-scan analysis

The full-body CT scan (64-row detector, LightSpeed VCT, General Electrics, Milwaukee, Wisconsin, USA) was performed in the supine position with a slice thickness of 0.625 mm, an interval of 0.625 mm, 120 kV and 200 mA in standard algorithm as previously described [3]. Additional three-dimensional and multi-planar reconstructions, as well as maximum intensity projections, were prepared on a workstation (ADW 4.4, General Electrics, Milwaukee, Wisconsin, USA). Certain reconstructed skeletal elements (femur, tibia, and humerus) were additionally used to calculate bone length and further parameters necessary for anthropological evaluation (see below).

### Autopsy

Since the full body CT scan provided some unclear data (see results), we decided to open the body. Furthermore, this direct approach allowed appropriate samples for all subsequent investigations to be obtained. In order to keep the body intact for another laying-out during the returning ceremony of the mummy, we applied a modification of present-day autopsy techniques. The reasons to do this, and the technical aspects of it, are presented in S2 File, which also summarizes major aspects of juridical and ethical considerations of our investigations.

Following the inspection of the skull, we decided to open the body from the dorsal side through two rectangular windows of chest and abdominal wall, on both sides of the vertebral column.

Therefore, the general´s uniform and his undershirt were opened on the dorsal side of the body (which was hidden after closure of the opening) by a sharp incision in an inverse T-shape along the uniform´s seam; the clothes were carefully separated from the mummy´s skin. Then the body was opened using an oscillating saw. Since CT scans had provided unclear images of the structures in the lower thorax/ upper abdomen and an “undefined lesion” along the right psoas muscle, the rectangular window on the right side was extended caudally to the posterior iliac spine (for details see S2 File). By this approach a precise and full restoration of the dorsal chest wall and the overlying clothes were easily achieved.

The internal parts of thorax and abdomen were then inspected directly or endoscopically, thereby allowing removal of pieces of soft tissue and obvious organ remnants for analysis. A ventral segment of the vertebral column was also removed for investigation and, more importantly, for sufficient material sampling further various subsequent analyses (see below). After completion of the examination, both dorsal windows were closed; the clothes were reapplied and repaired, and the mummy returned into its sarcophagus.

### Contact radiography

All removed major tissue samples or organs underwent contact radiography in order to identify areas of different radiodensity and, especially, calcification. These were done by routine plain radiological investigation (70kV). This was an indispensable aid to obtain precise samples for the subsequent histological and molecular investigations.

### Microscopic studies

Specimens obtained from several organs and tissues were processed for histological analysis. The preparation of the samples, the rehydration procedure, and the embedding and cutting techniques, have been previously described in detail [16]. Particularly, samples from both lungs (especially from areas with obvious calcification, but also from all different lobes), the heart, and the abdominal structures, were prepared. Particular attention was paid to the cystic retroperitoneal lesion on the right side. The sampling was precision-guided using the contact radiographs.

All histological samples were stained with H&E, connective tissue stains, PAS, Grockotts silver stain, Prussian blue and May-Grünwald-Giemsa stain, Ziehl-Neelsen acid fast bacilli stain, and Kongo red staining for amyloid deposition [16].

### Stable isotope analysis

Collagen was extracted from dentin (permanent tooth 25), rib and vertebral bone following well established methods [17]. Multi-element stable isotope analyses of carbon, nitrogen, sulphur and hydrogen on collagen samples were performed according to Lehn et al. [17].

Collagen samples were weighed in tin capsules (3x). Stable isotope ratios of carbon (^13^C/^12^C), nitrogen (^15^N/^14^N) and sulfur (^34^S/^32^S) were analysed by isotope ratio mass spectrometry (IRMS) at Isolab GmbH, Schweitenkirchen, Germany. The data are reported in *δ*-notation in per ml (‰) and measured relative to international standards (V-PDB for carbon, AIR for nitrogen, V-CDT for sulphur, and V-SMOW for hydrogen). The analytical uncertainties are 0.1‰ for *δ*^13^C, 0.2‰ for *δ*^15^N, 0.3‰ for *δ*^34^S, and 3‰ for *δ*^2^H.

The primary dentin of the second premolar develops from the 8^th^ to the 10^th^/12^th^ years of life, and is not subjected to any turnover for one’s entire life. Different to tooth dentin, bone constantly remodels during life. Collagen in rib and vertebra is thought, isotopically, to reflect an individual’s diet over about 6–10 years prior to death.

### Molecular investigation of ancient human and bacterial DNA

For further study of the individual, we applied ancient DNA tests in a two-fold approach (details of the molecular analysis and the reagents used are presented in S3 File). The first, a human genetic finger-printing assay, was performed in order to investigate any genetic relationship between Count Heinrich and the other burials of the Jordan crypt in Dötting/ Wackerstein. Therefore, bone tissue was used to extract human aDNA according to established protocols [4] Specimens from Count Heinrich LII. Reuss-Köstritz, Baron Friedrich Wilhelm von Jordan, his wife Violante, née Countess Sandizell, and their two children Baron Max von Jordan and Baroness Carolina von Jordan, were taken under the strict recommendations of contamination-free sample preparation for aDNA research [18]. The bone samples were milled, powdered and then the tissue powder was subjected to DNA extraction as described elsewhere [19]. After determining the amount of human DNA per sample using realtime PCR Quantifiler ^®^ Duo DNA Quantification Kit (Thermo Fischer Scientific) according to established protocols [20]. Individual STR-patterns were determined with the help of three different multiplex kits (Powerplex ^®^ ESX17 fast system, Promega; AmpFISTR ^®^ MiniFiler TM; AmpFISTR ^®^ NGM Select TM PCR Amplification Kits, Thermo Fisher Scientific) in an AB Viriti thermal cycler according to the manufacturer´s instructions, using 32 and 34 cycle programs. The PCR products were analysed on an AB 3500xL capillary electrophoresis system (Thermo Fisher Scientific) and data analysis was carried out using the GeneMapper^®^ ID-X Software v1.4 (Thermo Fisher Scientific). Consensus profiles were generated for each sample and compared between each other.

In the second approach, samples from each lobe of both lungs, bone, and the retroperitoneal cyst, were also subjected to aDNA preparation. Again, appropriate tissue samples were minced and powdered. DNA was extracted and amplified by PCR as done in established protocols [21]. The resulting aDNA was then used to identify the specific sequence of the MTC, IS6110, as performed previously [16] by gel electrophoresis and subsequent Sanger sequencing. Finally, further aliquots were used to identify substrains of the MTC by using the spoligotyping technique as previously described [21]. (Details of procedures and reagents are given in S3 File).

## Results

### External macroscopic aspects and anthropological estimations

In general, the mummy was well preserved and almost complete. Using the soft tissue preservation system (STPS) score [22] 22 out of a maximum 25 points was achieved i.e. a score value of 0.88. In this system the human body is divided into 5 anatomic body segments (head, thorax, pelvis, arms and legs) and each is assigned a maximum of five “points”. The examiner views the surface of the mummy and estimates the degree to which soft tissue covers the underlying skeleton. In the present mummy, only the skull region did not obtain the maximum point number resulting in the above indicated value.

Furthermore, the mummy permitted the following anthropological estimations: The complete body length was 165.5 cm and dry weight was 17.8 kg. Due to the post-mortal shrinkage, the complete body length was further calculated by radiological determination of the bone length using German standard reference values [23]. Thence, the femora indicated a whole-body length of 171.5 cm (right) and 172.5 cm (left), tibia (right) 164.5 cm and (left) 166.5, humerus (right) 174.5 cm and (left) 175.5 cm. These values were applied to the complex calculation model used by Schleifring et al. [24] to estimate the overall average value for the body height which resulted a mean value of 170.8 cm. Therefore, a post mortem body shrinkage of ca. 5.3 cm could be calculated.

Further evaluation of the humerus-length-index indicated right-handedness (right 19.59 vs. left 17.23), and of the femur-robusticity-index a slight, but similar preference of use, of the right lower extremity (right 9.87 vs. left 9.72) and the index platycnemius ranges within “eurymere” values suggesting a moderate walking activity [25].

The further inspection of the mummy revealed poor dentition with only one tooth preserved on each side of the maxilla (d13 and d25) and three teeth on both sides of the mandible (d31/d33 vs. d41/d43). All teeth show severe dental attrition (Perizonius and Pot grades 3–5+ [26]) with two teeth (one on each side of the mandible) broken with a smooth surface. All teeth revealed either occlusal and/or approximal caries. However, there were no periapical abscesses or periodontal disease. All other teeth were lost pre-mortem, as seen by complete closure of the alveolar cavities.

### Palaeoradiological CT-investigations

The further detailed whole-body CT-scan confirmed the aforementioned details, adding significant internal findings. In general, the application of the previously described CT-based soft tissue preservation score (STPS) [27] revealed quite well rankings. In this system, the score assigns specific values for a series of defined radiological checkpoints, each divided into specific subcategories, e.g. for the organ systems evaluating the central nervous system (10 points), cardiorespiratory system (20 points), gastrointestinal system (40 points), genitourinary system (20 points), vascular system (10 points). For each subcategory the presence/absence of a specific structure determines the resulting value which is then added to the overall score value. The present mummy thereby achieved 97 out of 100 points (97%) for soft tissues of head and musculoskeletal system (score A), and 67.5 out of 100 points (67.5%) for organs and organ systems (score B). Accordingly, the CT-based soft tissue score is in a similar range to the macroscopic STPS score.

#### Skeleton

**Fig 4:**
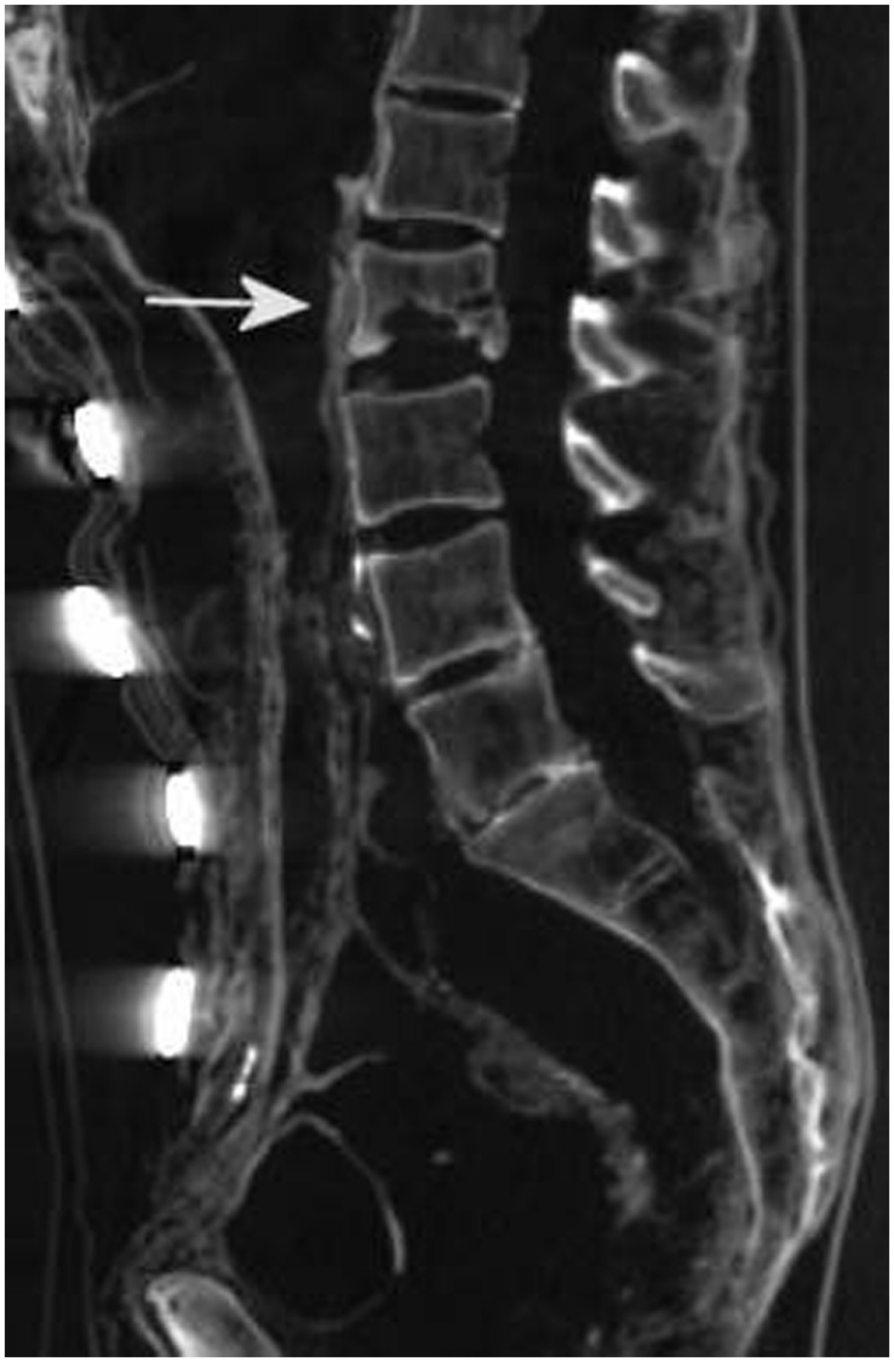
CT scan of the lumbar vertebra showing a localised irregularly-shaped osseous defect of L2 (arrow).

In general, the skeleton was excellently preserved and the bone substance was considerably well structured without any evidence of age-related osteopenia. Besides minimal and obviously post-mortal dislocations of the cranio-cervical and C2/3 segments of the vertebral column, the second lumbar vertebral body revealed major irregular osteolytic destruction with sclerotic bone reaction (max. 10 mm in size) (Fig 4). Beyond this lesion several other vertebral bodies showed partly bridging osteophytosis and synostosis, as typically seen in diffuse idiopathic skeletal hyperostosis of the vertebral bodies (DISH, M. Forestier). In line with these findings there was interspinal bridging of the vertebral bodies with sclerotic bony structures (slightly more pronounced on the left than the right), however, without significant narrowing of the nerve canals.

The other joints showed variable signs of degenerative joint disease (arthrosis) with mild degeneration of both shoulders and the right hip joint, and moderate degeneration of the sternoclavicular joints and the symphysis. All other joints, in particular the small joints of hands and feet, but also both ankle and elbow joints, remained free of arthritic degeneration. Only, the right distal talus contained a small (12 mm) osteolytic zone in the subchondral bone with sclerosis, consistent with an intraosseous ganglion. The same talus showed a small osteochondral lesion, such as in a post-traumatic condition, on its medial side.

Finally, both big toes had hallux valgus deformity with associated hyperextension of the metatarsophalangeal joints and hyperflexion of the distal interphalangeal joints.

#### Neck and thorax

The left thyroid gland was significantly enlarged (size 70 x 43 mm) with focal, and somewhat nodular, transformation, and focal calcifications up to 10 mm size, thus representing the features of a regressively transformed *struma nodosa*. This caused slight deviation of the trachea to the right side but without compression.

**Fig 5:**
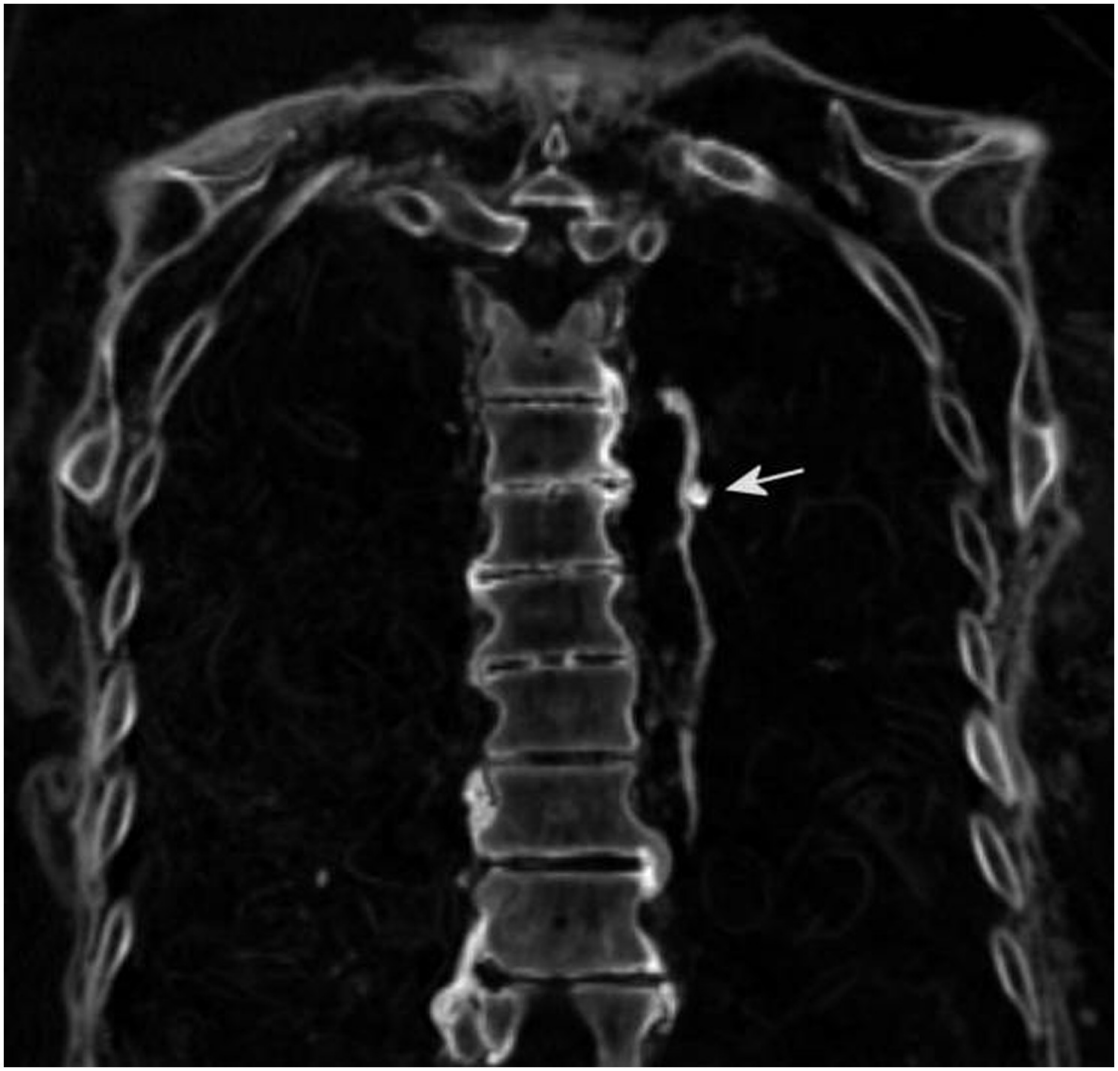
Sample of the CT scans of the lungs: Here the retracted left lung shows hilar calcifications (arrow) and significant osteophytosis of the vertebrae.

Within the thorax, the lungs were significantly shrunken and obviously retracted with residues along both sides of the vertebral column. Both lungs contain small calcifications (3–8 mm) affecting the pulmonary periphery bilaterally, but also including the lung hilae (Fig 5).

Additionally, the thorax contained the pericardium and the shrunken remnants of the heart muscle. The latter showed significant streak-like calcifications along all three functional cardiac blood vessels; these calcifications were in a double-track pattern. There was no evidence for vascular luminal obliteration (see below).

#### Abdominal cavity

**Fig 6:**
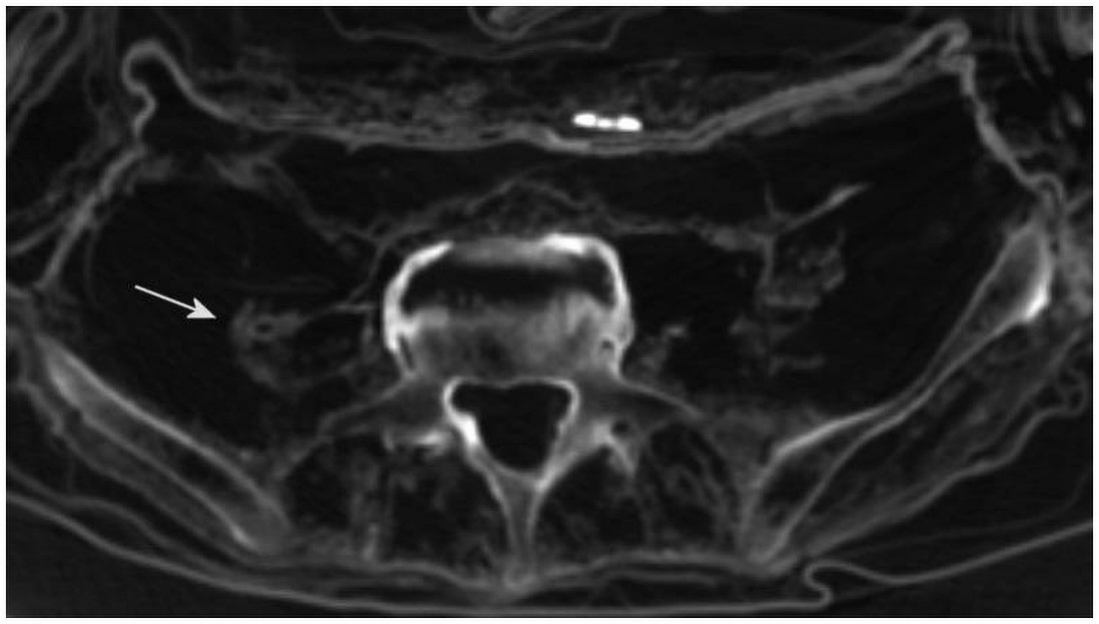
CT scan of a retroperitoneal mass on the right side (arrow).

**Fig 7:**
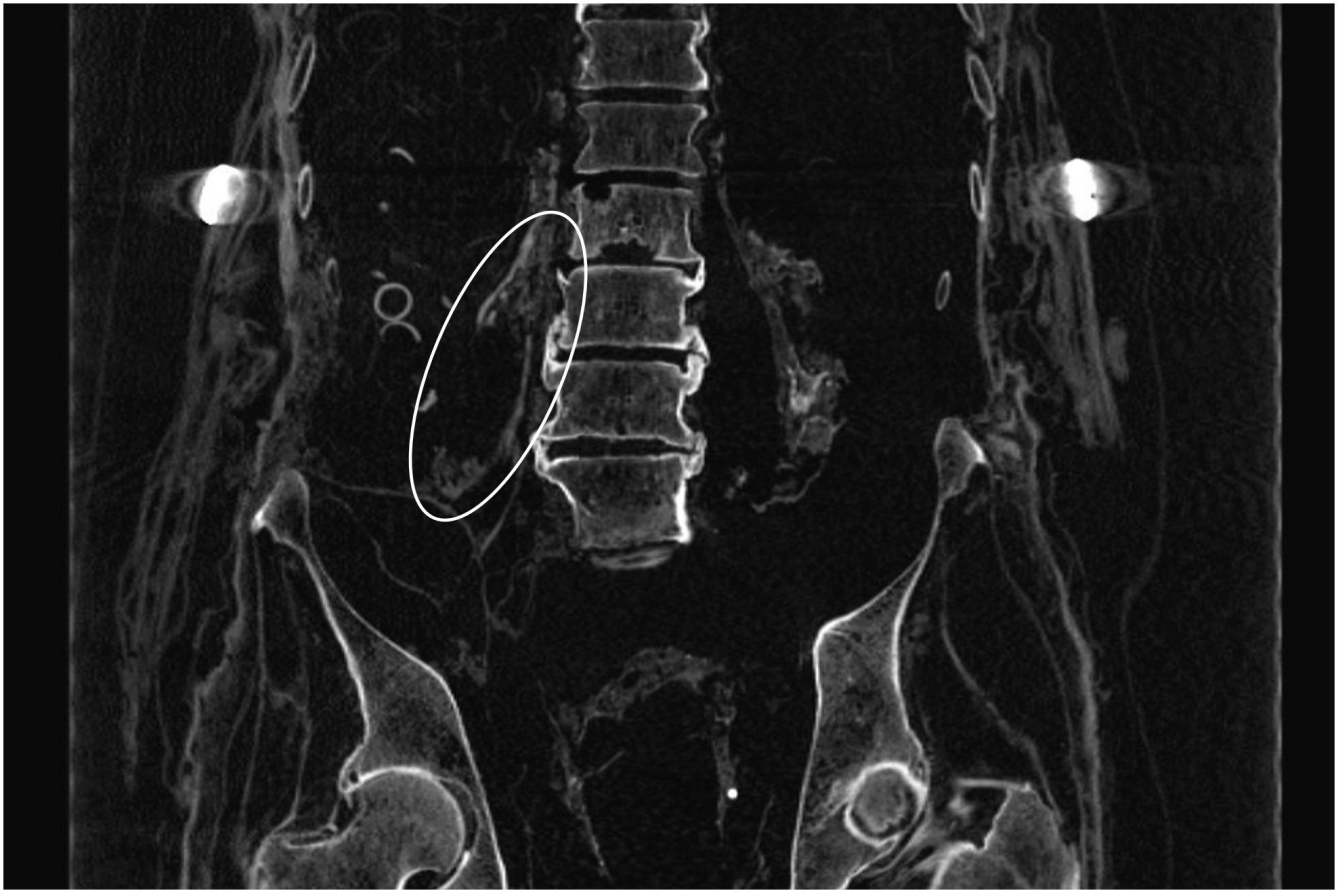
Coronary plain section of the CT scans with a further aspect of the cystic lesion at the retroperitoneal space (circle).

The CT-scan revealed an unusual condensed structure at the right retroperitoneum with a central cyst-like disaggregation reaching approximately 8 cm length and up to 5 cm width (Figs 6 and 7). This structure extended along the right psoas muscle and contained occasional small streaky calcifications. Adjacent structures, such as the urinary bladder and the rectum, remained unaffected. There were some small calcifications of the phlebolith type. The penis and scrotum are unremarkable. Otherwise, the abdominal cavity was void of organ structures. However, the transition zone of the thorax to the upper abdomen on both sides revealed several shell-like, dense structures with irregular margins and a very irregular distribution pattern. These structures could not be attributed to any organ or organ remnant and therefore their origin remained unclear.

#### Vascular system

**Fig 8:**
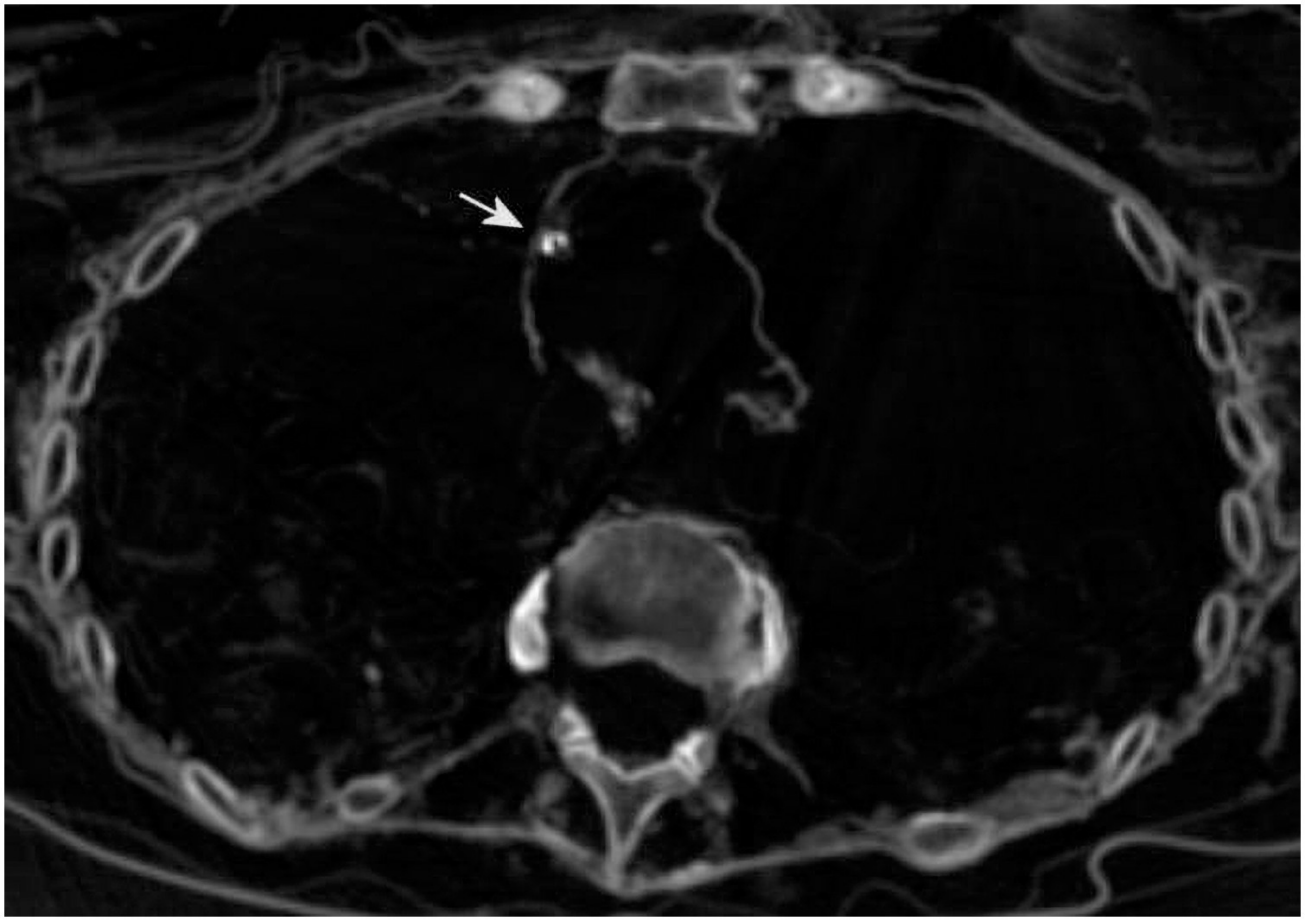
CT scan of the heart showing focal ring-like calcification of one of the coronary vessels (arrow).

**Fig 9:**
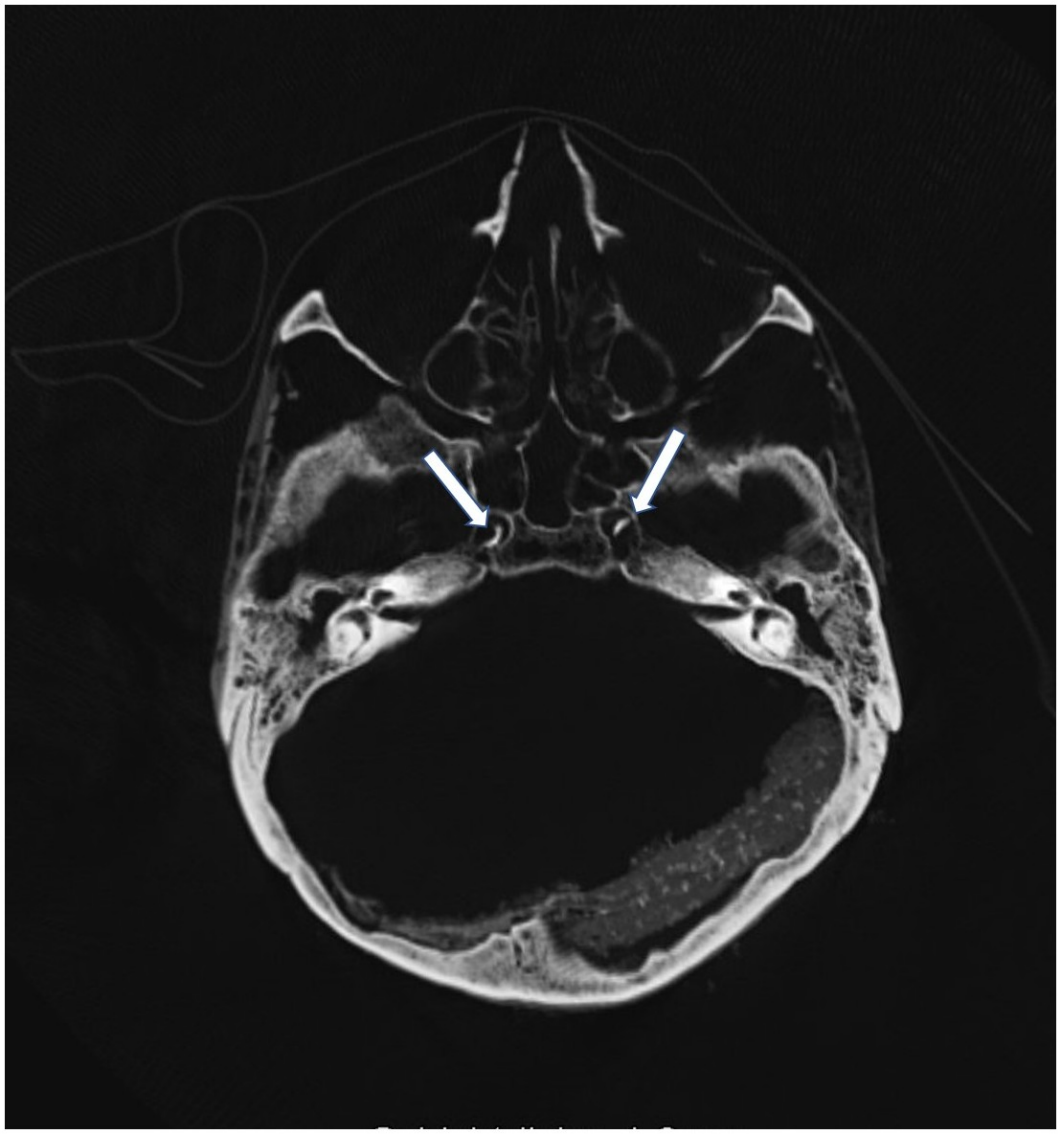
CT scan of the skull base showing bilateral calcifications of the internal carotid artery (arrows).

**Fig 10:**
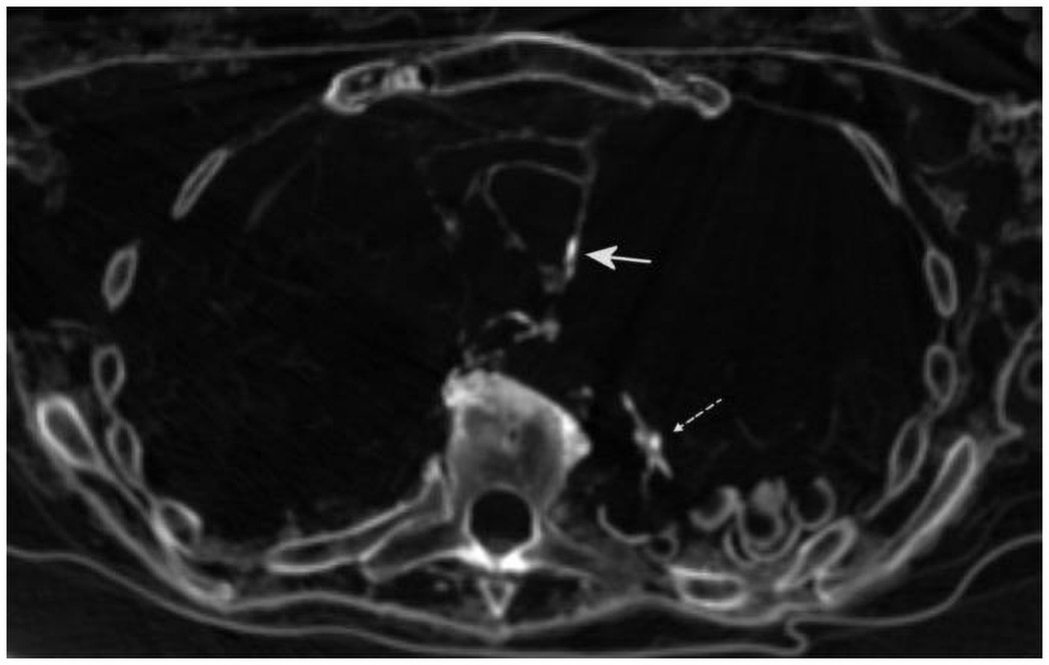
CT scan cross section through the thorax showing focal calcifications of the vessel wall (full arrow); note the peripheral pulmonary calcifications (dotted arrow) that occurs occasionally in this cut of the left lung. Additionally, the shell-like irregular objects are clearly visible.

Besides the arteriosclerosis of the cardiac vessels (Fig 8), similar calcifications were detected in both inner carotid arteries (both in the extracranial and the endocranial part, Fig 9). Focal arteriosclerotic calcifications were also detected along the thoracic (Fig 10) and abdominal aorta, both inner iliac and femoral arteries, and the popliteal arteries, however without evidence of major stenosis of the vascular lumen.

### Autopsy findings

**Fig 11:**
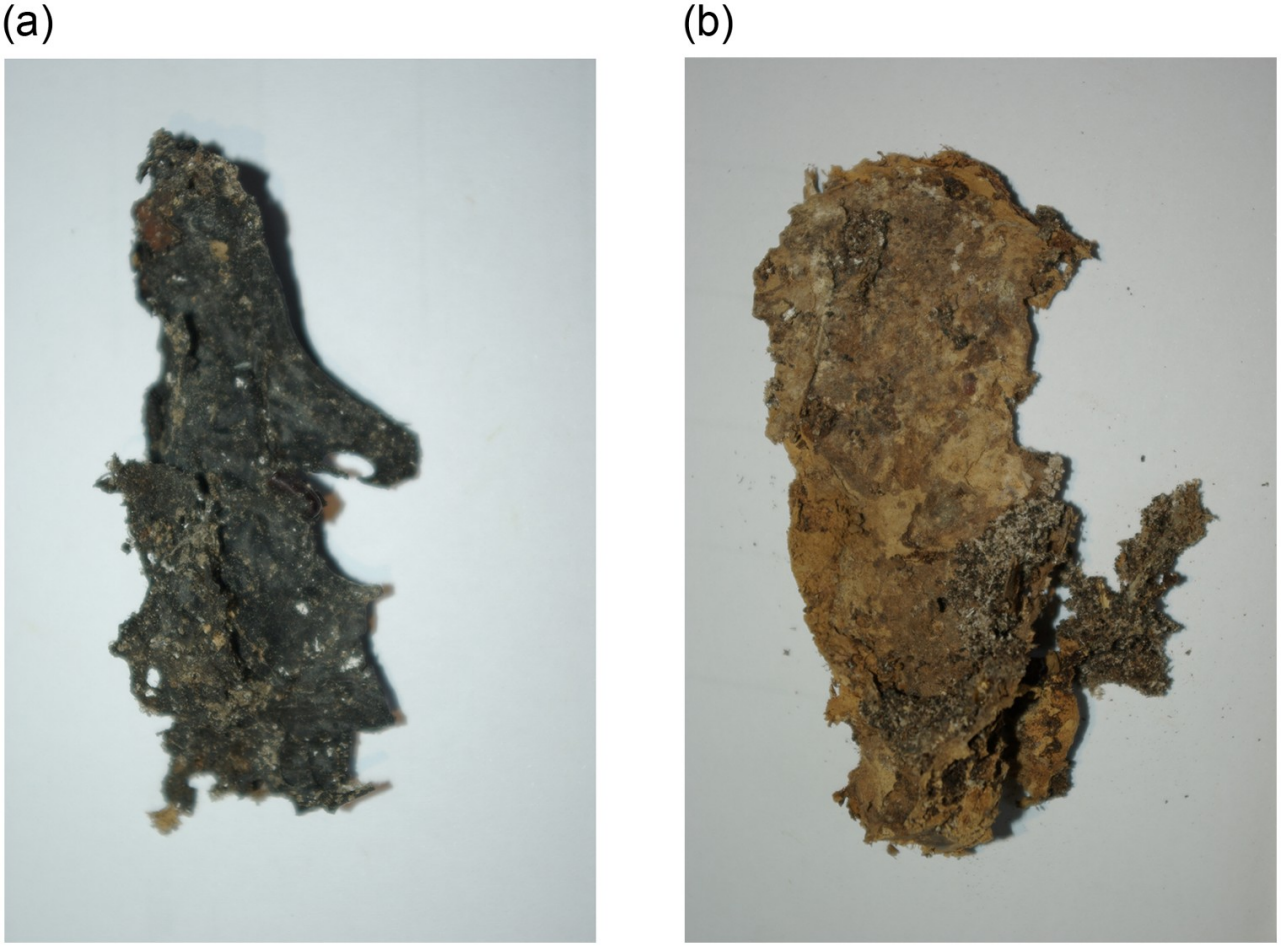
The autoptically removed right (A) and left (B) lung—Macro-preparation.

Since the whole-body CT scans had shown several unidentified objects in the lower thorax and upper abdomen of both sides and the lower abdomen of the right side, we decided to perform a mummy autopsy. Following the opening of chest and abdomen from the dorsal side, we were able to identify both lungs, the pericardium and the shrunken heart, which concurred with the CT-scan observations. Both lungs were significantly shrunken and retracted to the dorso-medial thorax presenting as flat organ residues (Fig 11). Furthermore, the opaque irregular structures in the chest and the abdomen, were easily identified as nut-shells that obviously had been introduced post mortem into the body by some rodents whose faeces were also found within the body in small amounts.

**Fig 12:**
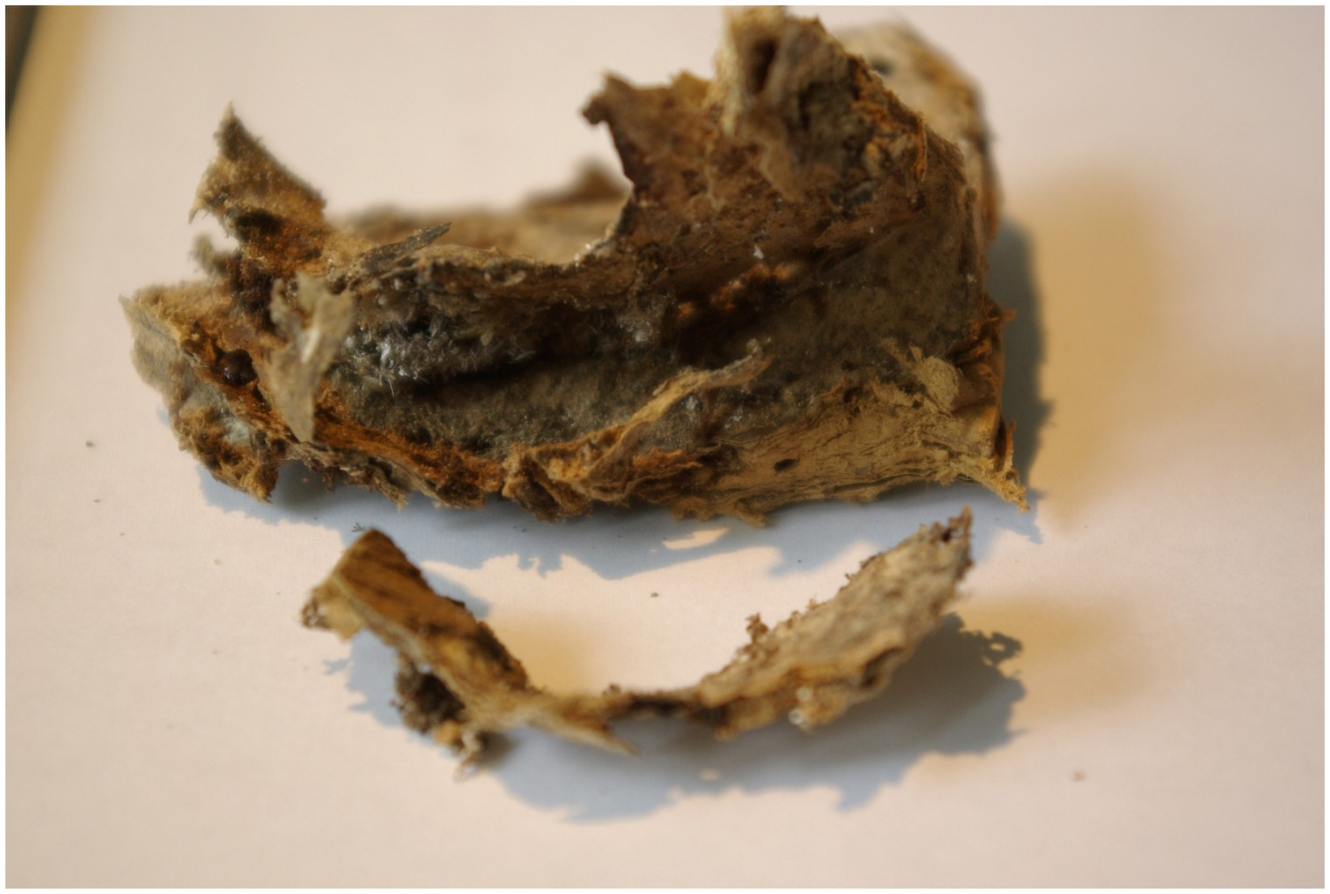
Macro-preparation of the autoptically removed retroperitoneal cyst. The cyst has been incidentally opened during removal from the body.

Subsequent to the opening of the abdominal cavity, the right retroperitoneal mass presented as a central cyst with a surrounding dense capsular connective tissue wall (Fig 12) extending along the right retroperitoneal space down to the pelvis. Beyond these structures, the abdomen contained only connective tissue bundles of the diaphragm and some residues of the small and large intestine, but no remains of identifiable organs such as liver, spleen or kidneys. Furthermore, the unidentified objects in the CT scans were identified to represent nut-shells that had been brought into the body obviously from the surroundings—possibly by some rodents that had obtained access to the coffin and the inner parts of the mummy via the anal canal.

Finally, three continuous vertebral bodies (ventral part) were removed, particularly in order to obtain sufficient material for subsequent extensive further analysis. Removal of the posterior elements of the vertebral column as well was not undertaken in order to conserve the structural integrity of the mummy, and avoid deforming collapse when handling it during reburial.

### Contact radiography of removed organs and tissue samples

The removed organs and tissue samples were furthermore subjected to direct radiography. This was necessary in order to allow the exact removal of samples for subsequent histological and/or molecular investigations, a particularly important approach that ultimately provided us with a very precisely located series of samples for the study of regional differences in the pulmonary pathology which would have been impossible to detect by CT-scans alone.

Accordingly, this approach confirmed the small calcifications in both lungs as found on CT-scans with a major calcification area in the right lung centrally, and several specks in the periphery of both lungs.

The retroperitoneal cyst also contained flake-like calcifications most obvious in its caudal part, but was otherwise without major calcium deposits.

The heart was remarkable since all three functional coronary arteries revealed streaky calcifications along their course, but there were no calcifications within the obvious remnants of the heart muscle.

Finally, the bone of the vertebral column, as well as some smaller segments of dorsal ribs that had also been retained, showed dense bone tissue fairly unexpected for the individual´s old age. However, the bridging spondylarthrosis seen in the CT-scans of the vertebral column was easily visible on the contact radiographs. This is consistent with Diffuse Idiopathic Skeletal Hyperostosis (DISH).

All contact x-rays are presented in a supplementary file in the S4 File.

### Histomorphology of the internal organs

Additionally, we performed histopathological investigations of most of the removed organs and tissue samples which, in most instances, were well-preserved structures.

**Fig 13:**
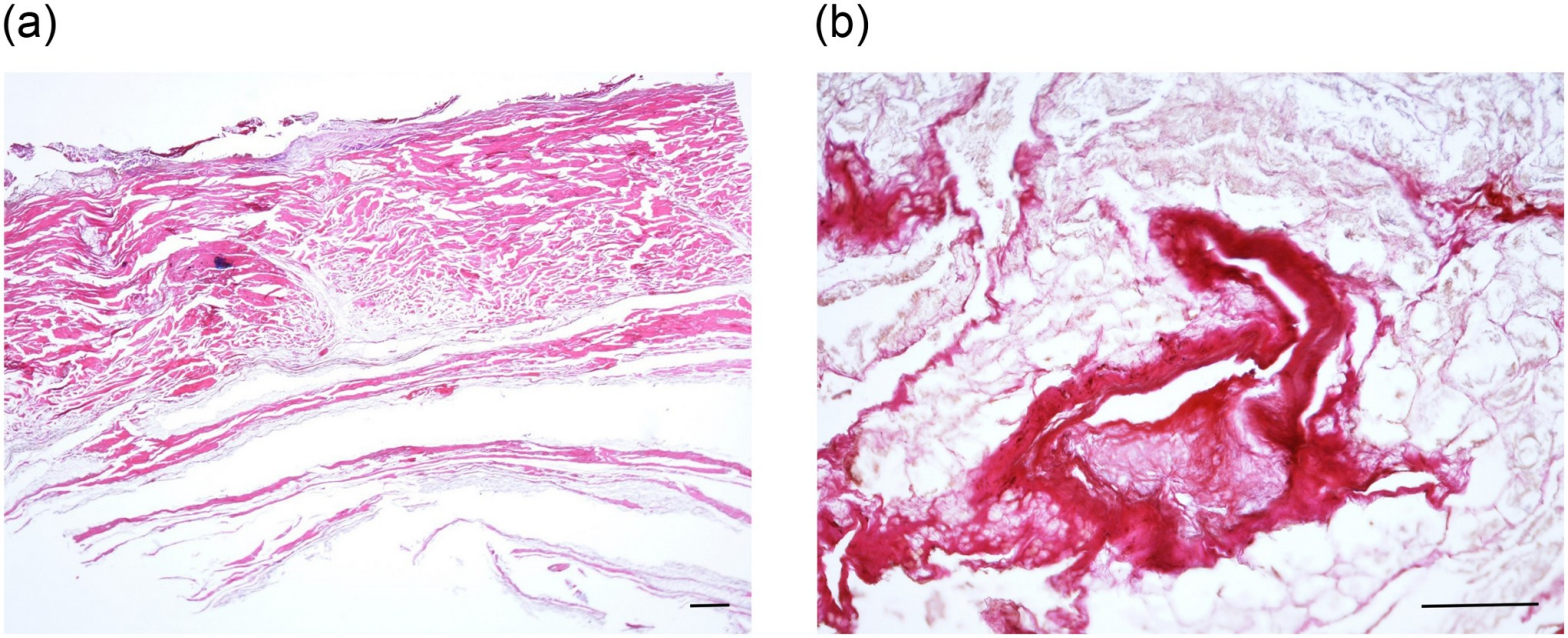
A. Histological section through the mummy’s skin. Despite the loss of the superficial epidermis the collagen matrix of the dermis is excellently preserved. (H&E); bar = 1.000 μm. B. Histological view of a small arteriole of the subcutaneous skin tissue of the mummy. Again, very well-preserved structures are present. There is no evidence for vascular narrowing (microangiopathy) (Elastica-van Gieson stain); bar = 200 μm.

Likewise, despite the obvious lack of the superficial epidermis, the mummy’s skin was easily recognisable (Fig 13A). The connective tissue of the dermis, small arterial blood vessels (Fig 13B) and subcutaneous fat were routinely present and revealed no pathological change, in particular there was no evidence of systemic pathological deposition, such as by amyloid or mucoid substances. However, as expected, no cellular details were preserved.

**Fig 14:**
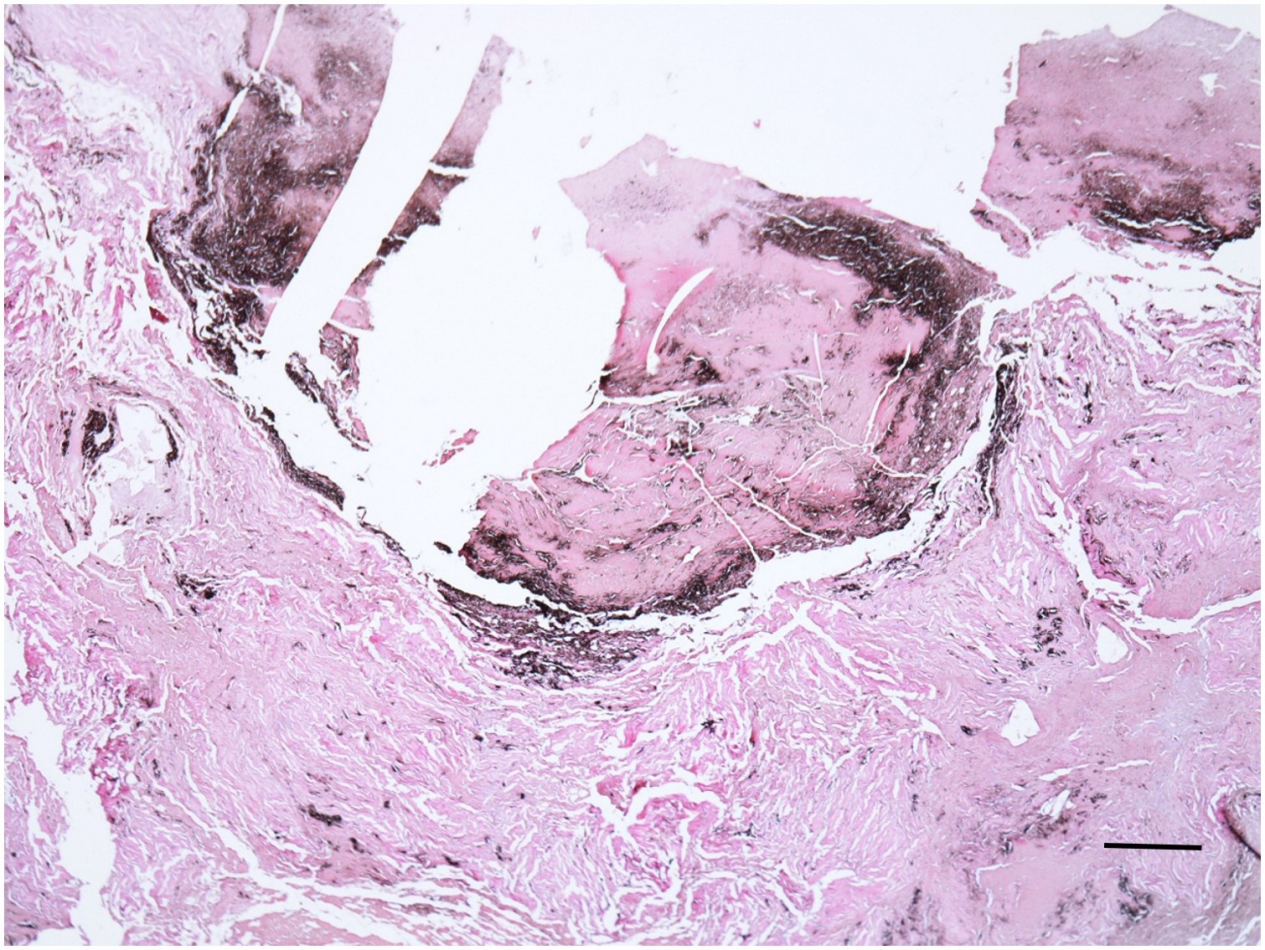
Histological section of hilar nodular condensation of the right lung showing scar-like fibrosis and significant anthracosis. Despite decalcification prior to the preparation there are unfortunately some artefacts from the calcifications (H&E); bar = 200 μm.

**Fig 15:**
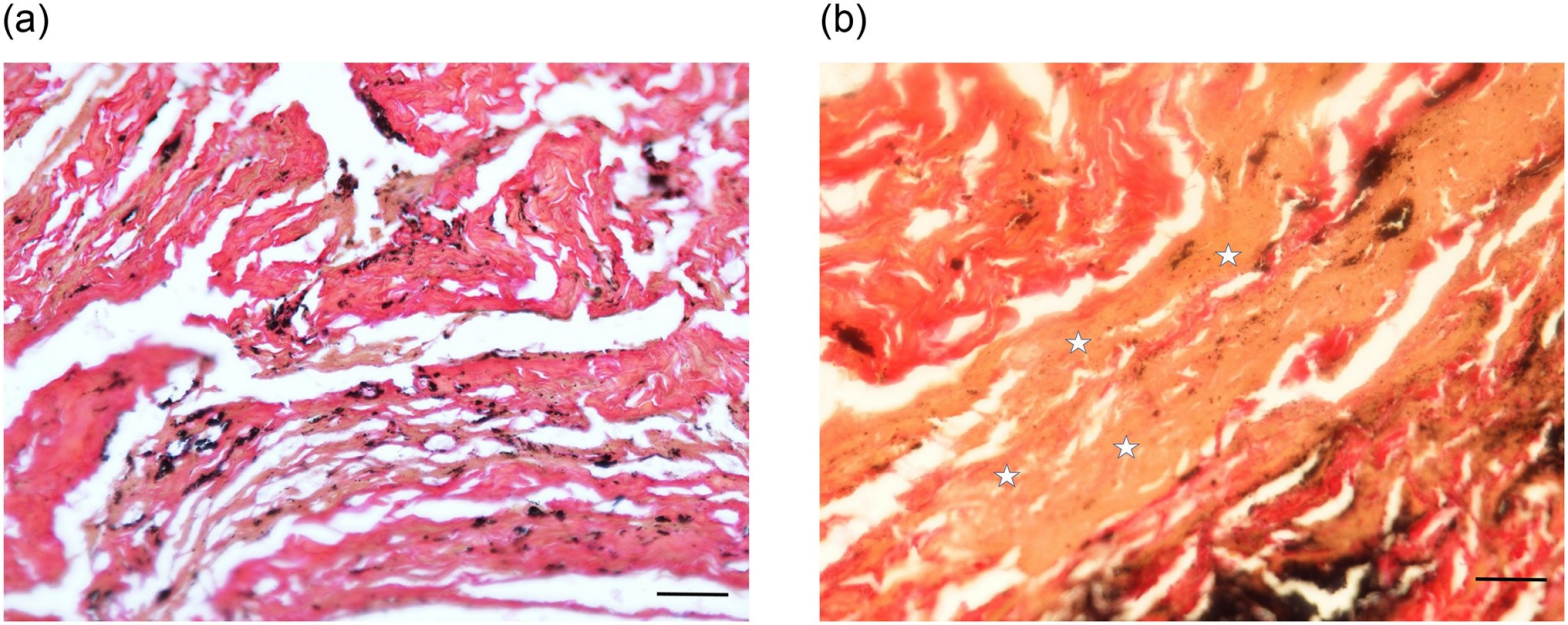
A: Histological section of peripheral lung parenchyma of the right upper lobe showing post mortem condensed lung alveoli. (H&E); bar = 30 μm. B: At the right lower lobe a comparable histological section to Fig 15A reveals extensive intra-alveolar proteinaceous exudation (yellow material) (Elastica-van Gieson stain); bar = 30 μm. The stars indicate the condensed intraalveolar protein material.

Several histological samples were obtained from the lungs, both from the central and the peripheral regions. Most remarkably, the right central lung showed a circumscribed scar-like nodule with calcifications, significant anthracosis and scar-like fibrosis (Fig 14). On special staining however, neither acid-fast bacilli nor parasites nor fungi were detectable. In the lung periphery, the alveoli were densely collapsed, but still discernible (Fig 15A), while in the left lower lobe an obviously proteinaceous exudate seemed to fill part of the collapsed alveolar spaces (Fig 15B). Again, special stains showed neither specific microbes nor haemosiderin deposits nor amyloid deposition. Cellular details, of course, were again completely absent.

**Fig 16:**
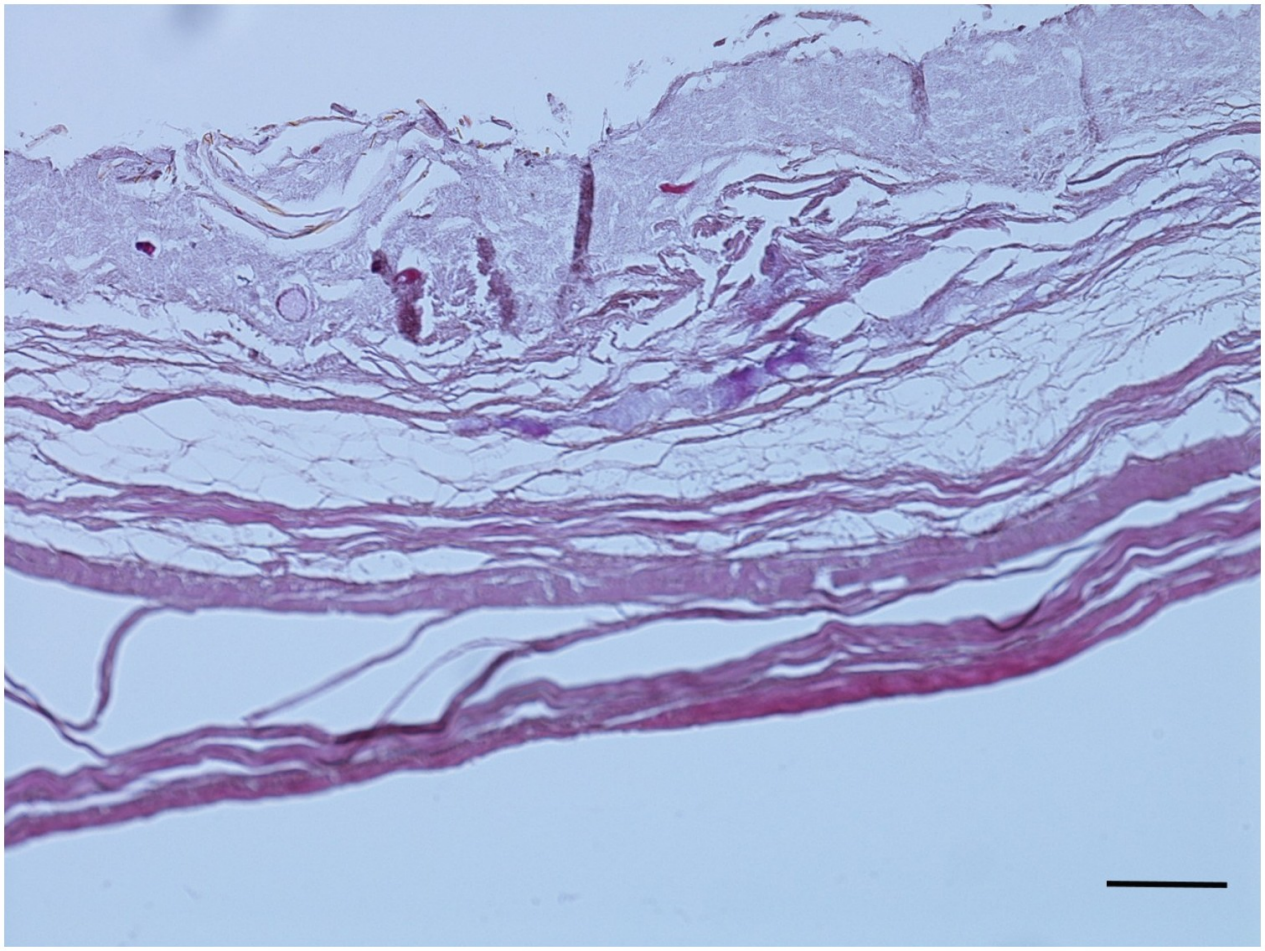
Histological preparation of the cystic wall showing a pseudocapsule and amorphous inner material. (Elastica-van Gieson); bar = 200 μm.

**Fig 17:**
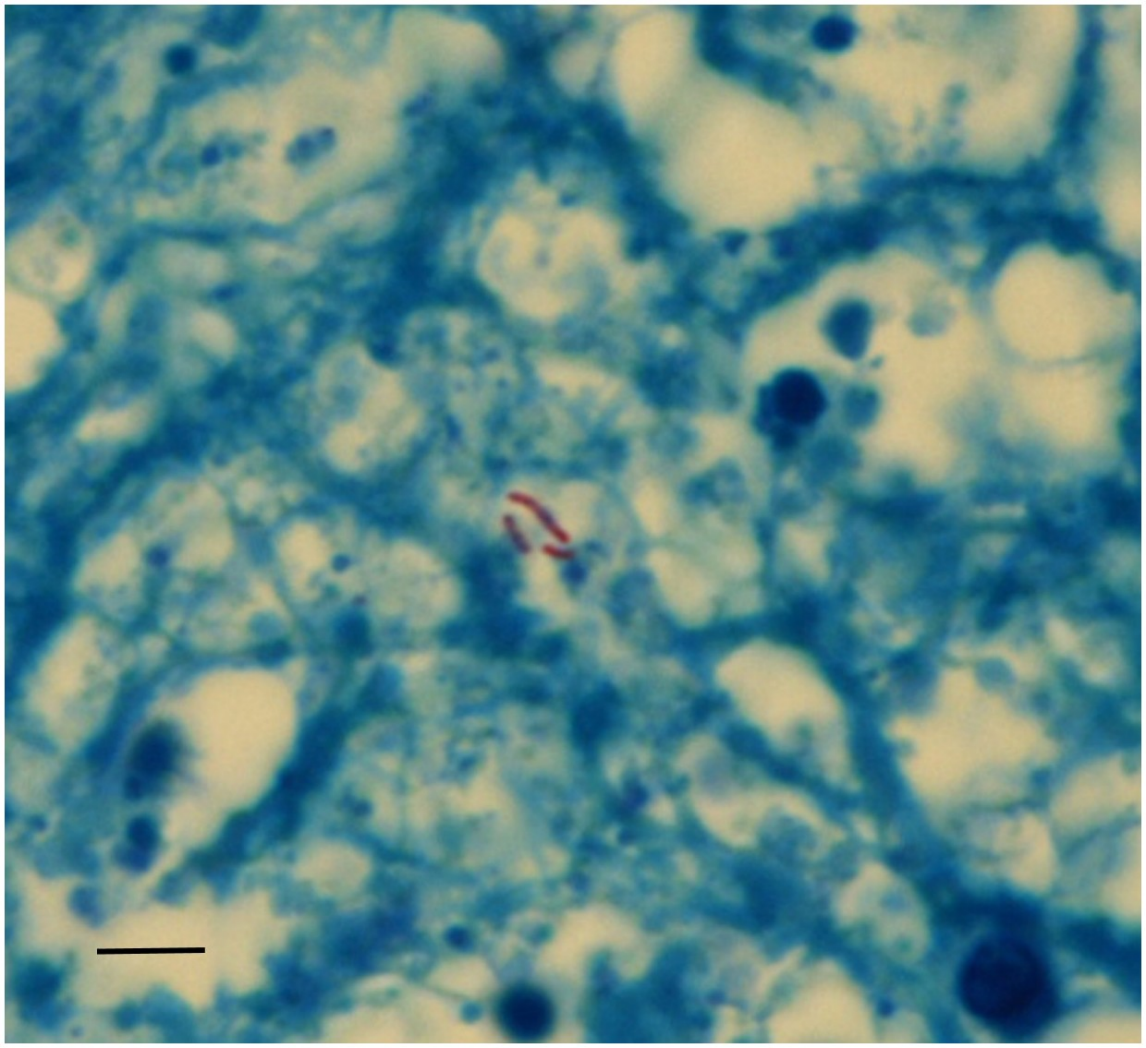
Ziehl-Neelsen special stain for acid-resistant bacilli shows a few positive rod-like mycobacteria in the amorphous inner layer of the “cyst wall” (Ziehl-Neelsen-staining); bar = 10 μm.

Most remarkable were samples prepared from the retroperitoneal mass, adjacent to a capsular connective tissue, a broad band of amorphous detritus was seen containing small corpuscular structures (Fig 16), with occasional acid-fast bacilli (in Ziehl-Neelsen stain) (Fig 17). All other stains remained negative i.e. no evidence of old healed bleeding residues, parasites, fungi or amyloid deposits.

Samples from all other organs and tissues, such as the heart muscle, were less well preserved and did not show pathological changes.

### Stable isotope pattern

Stable isotope analyses of carbon, nitrogen, sulphur and hydrogen were performed on collagen samples of dentin, rib and vertebral body bone (Table 1). The collagen extracted from ground dentin and bone material followed the collagen quality criteria [28,29].

**Table 1:** Stable isotope values of carbon, nitrogen, sulfur and hydrogen in collagen samples from tooth and bone tissue of Heinrich LI. Reuss-Köstritz.

	yield of collagen (%)	C/N ratio of collagen	*δ*^13^C_VPDB_ [‰]	*δ*^15^N_AIR_ [‰]	*δ*^34^S_VCDT_ [‰]	δ^2^H_VSMOW_ [‰]
tooth dentin*	15.9	3.2	-19.2	12.9	6.3	-44
vertebra bone	22.7	3.2	-19.0	12.5	7.1	-30
rib bone	2.,2	3.2	-19.1	12.6	7.2	-35

*(premolar region 25).

The analytical uncertainties were ±0.1‰ for δ^13^C, ±0.2‰ for δ^15^N, ±0.3‰ for δ^34^S, and ±3‰ for δ^2^H.

The isotope signatures of the body tissues represent Count Heinrich’s dietary habits during his childhood up to his 12^th^ year (molar sample) and the last 6 to10 years of his life (vertebra and rib samples). The three collagens, although representing various time ranges of metabolism, revealed almost similar isotope values for the elements. The somewhat lower stable isotope values for sulphur and hydrogen in tooth dentin may be tissue dependent.

Stable isotope values for carbon show a diet based on C3 plants (e.g. wheat, rye, barley) that was common in Central Europe. Stable nitrogen isotope values reflect the intake of a relatively high amount of animal protein, at least during Count Heinrich’s childhood and at an advanced age, which may indicate a high social status and a luxury diet. Stable sulphur isotope values vary depending on the consumption of seafood and on the geological conditions. Finally, stable isotope values for hydrogen primarily represent the local climatic conditions. The *δ*^34^S values in the collagens show a diet mainly based on food products from terrestrial animals.

The isotope signatures correlate well with the typical diet of contemporary South German noblemen, indicating a high consumption of terrestrial animal protein, at least during his childhood and at an advanced age [30].

## Ancient DNA analysis of human DNA

The molecular investigation of the individual´s genetic pattern followed the current routine tests for paternity in forensic settings. Due to the excellent preservation of the ancient biomolecules, an almost complete STR-profile could be obtained from the sample from Count Heinrich. All 20 STR-markers were seen in that from Baron Wilhelm von Jordan, his wife and his son, and a partial but still meaningful profile was retrievable from the mummy of their little daughter Carolina von Jordan (for the results see S5 File).

There was an absolute proof that Heinrich LII. Reuss-Köstritz was not genetically related to any person from the crypt owner´s family.

### Ancient DNA analysis of the *M*. *tuberculosis* complex

**Fig 18:**
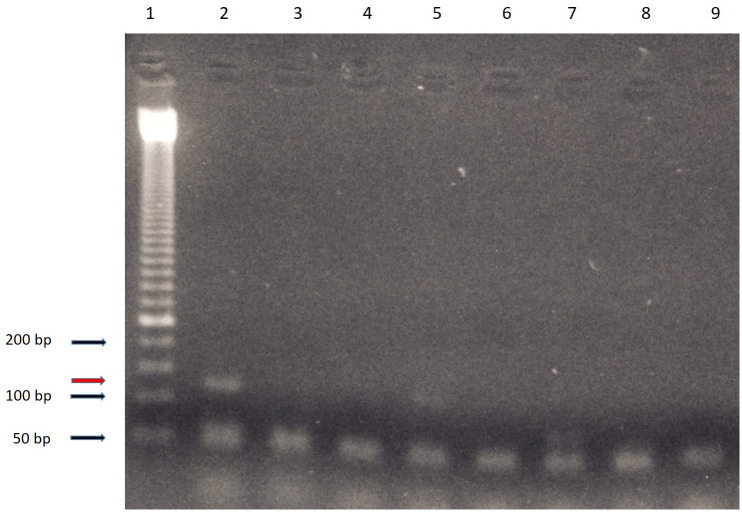
Gel electropherogram of the amplification products of the mummy´s tissues. 1 size ladder; 2 sample of the cyst wall; 3–6 various samples from both peripheral lungs; 7 sample from the right pulmonary hilus (calcified nodule); 8 sample from the L2 vertebral body; 9 negative control. The amplicon sizes are indicated in the left (dark arrows); the target amplicon size of 123 bp is shown by the red arrow (left).

In order to further prove the bacterioscopical evidence for a chronic tuberculous abscess at the psoas muscle, we further extracted aDNA from the cyst capsule and searched for the *M*. *tuberculosis* complex using the widely applied sequence IS6110. A typically positive amplicon of the expected size was detected in a sample from the cyst wall while other samples from both lungs, including the right hilar mass, and the L2 vertebra, remained negative (Fig 18). Furthermore, all the negative and blank extraction controls run in parallel remained negative. In a further attempt at molecular characterisation, the amplicon was subjected to Sanger sequencing and showed 100% matching quality to the known MTC sequences (query coverage 100%, gaps 0%, no mismatches).

In parallel, samples from various regions of both lungs, including the right hilar mass, the lumbar spine and heart muscle, were investigated as well. None of these samples yielded a positive result in terms of MTC aDNA.

**Fig 19:**
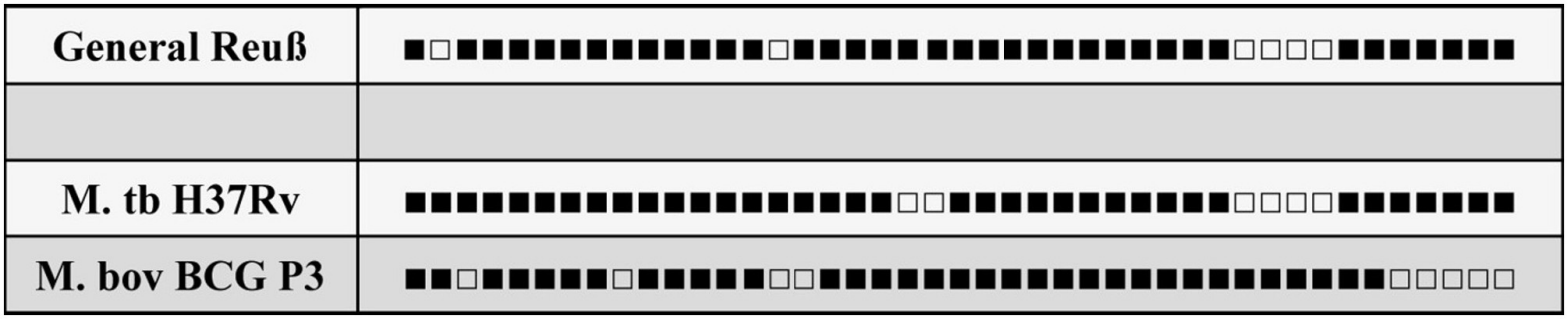
Spoligotyping results of the mycobacterial aDNA of the cyst wall.

Finally, the extracted aDNA of the retroperitoneal cyst was subjected to a spoligotyping analysis as previously applied to mummy material. Again, a specific pattern was detectable (Fig 19) with a highly comparable result to the standard reference sequence of *M*. *tuberculosis* H37Rv strain which is mostly used as reference. In contrast, the spoligotyping pattern was significantly different from *M*. *bovis* (strain BCG P3) and other members of the *M*. *tuberculosis* complex.

## Discussion

Any multidisciplinary study of human mummified corpses may unravel features of an historic person´s life, disease and/or death. The “outcome” of such studies strongly depends on the material with respect to the tissue available (skeleton vs. soft tissue/ internal organs) and the state of tissue preservation.

In addition to the anthropological-biomedical observations, the reconstruction of the life of a specific historic individual depends on the availability of historic data, such as official documents, letters or indirect literary evidences. Count Heinrich LII. Reuss-Köstritz fortunately was a person in public life which means that there exist significant direct (e.g. letters) as well as indirect (e.g. newspaper reports) information about him. Besides the reconstruction of his life, living conditions and diseases, this allows some correlation between the biomedical-anthropological observations and the reported facts of his life.

### Some juridical, ethical and technical aspects of mummy studies

Before the aforementioned results of the extensive biomedical study may be discussed in detail, a brief view may shed some light on major juridical, ethical and some technical aspects of mummy studies in general (a detailed discussion is given in S2 File). Juridical aspects may vary from country to country. In Germany, generally the land owner of the burial site (cemetery, crypt etc.) is responsible for the human remains; most cemeteries are operated by the local community authorities and are therefore in public possession. However, particularly older cemeteries (often associated with a church), may still be in the hands of the church. In both instances, manipulations of tombs and crypts, and the investigation of eventual human remains are dependent from the consent of the respective owner (such as in our case the Diocese Regensburg).

The scientific investigation of human mummified remains is a centre piece of human anthropology and paleopathology. The scientific value of both disciplines is beyond of any doubt. However, all studies require a respectful ethical handling of those human remains that should balance scientific “curiosity” with the optimal way to preserve those remains. Furthermore, the ethically “correct” way of handling is often dependent from local religious and ethical standards that may differ widely all over the world. In Central Europe the ethical standards are significantly influenced by the recommendations of the various Christian churches. These clearly permit scientific investigations, particularly when these contribute to the overall knowledge to the benefit of mankind.

It is clear that in every investigation, the extent of damage should be kept as low as possible. However, it is also without doubt that not all issues can be solved by even the most modern non-invasive analytical techniques. Likewise, in the present study neither the nature of the unclear cystic structure nor the irregular shell-like objects in thorax and abdomen would have been identified. Also, the histological identification of local processes can only be solved by sufficient material which may not be retrieved by minimal approaches. In the present case we choose a step-wise technical investigation which, however, permitted to retain the complete and intact outer appearance of the body.

### Life and living conditions of Count Heinrich LII. Reuss-Köstritz

We know from written sources many details about events during the life of Count Heinrich LII. Reuss-Köstritz. Data directly relevant to his health conditions, however, are limited. In particular, we have no medical reports or other direct health data beyond his death certificate. Nevertheless, in a few personal letters, Count Heinrich or his correspondents, describe health-relevant events and even describe diseases (cf. S1 File).

Despite these limitations, we are able to correlate physical data with his history. Count Heinrich died at the age of 88 years. A thorough investigation of the corpse (especially by complete body CT-scan) revealed no evidence for major (osseous) trauma sequelae which is well in line with the reports of his life where there exists no indication of any significant injury, despite numerous military campaigns (for detailed data on the life history the reader is kindly referred to Count Heinrich´s biography which also presents all known details as to his military and extra-military activities and events during several Napoleonic wars and the post-Napoleonic period in the Kingdom of Bavaria [15]).

The structure of his bone tissue was very healthy without any evidence of significant age-associated loss in bone mass (osteopenia). This seems unusual since age-associated osteopenia (which may clinically manifest as osteoporosis [31]) is a frequent disease in this age group with a current prevalence of clinically relevant disease in 45–55% of males of more than 80 years [32,33]. However, the stable isotope pattern suggests a continuously well balanced diet which can be attributed to the personal wealth of Count Heinrich. He died with a considerable fortune of approximately 150,000 (Bavarian) guilders (fl.) [15] around 4.5 million € today [34]. Only the post-mortal dental status indicates, from the last period of his life, limitations in his food consumption, since only a few mandibular and isolated maxillary teeth were present, and most other teeth had gone lost prior to death. The presence of dental caries indicates considerable carbohydrate consumption. In general, however, the nutritional supply of Count Heinrich must have been quite well balanced.

Furthermore, written evidence indicates that his physical activity was high even until advanced age [15]. Likewise, he presided over the local bible club until a few months prior to his death. He was only excused from this duty when he accompanied members of the royal family on numerous trips and journeys, until the last year before his death. Therefore, this high activity level coupled with adequate nutrition, are well in line with the excellent bone structure even at an age of 88 years. This activity must furthermore have been balanced since most of his large, weight-bearing joints did not show significant arthritic degeneration; accordingly, we find no evidence of physical overload, e.g. by continuous and/or focally enhanced activities. Again, this feature is very well in line with written evidence that indicates significant mobility up to old age.

Besides these features, this study, however, also identified specific diseases.

### Evidence for cardiovascular disease

The whole-body CT scans clearly showed calcifying arteriosclerosis. This affected the aorta and also the smaller arterial blood vessels, particularly those of the coronary and carotid vasculature. Likewise, we found several small calcifications in the vessels of all three functional coronary arteries. This was further substantiated by plain radiographs of the autoptically removed heart. However, the heart muscle itself remained free of calcifications, and there was no histological evidence for significant cardiac fibrosis. Accordingly, there was no evidence of a major old-healed myocardial infarction.

Similar arteriosclerotic plaques were detected radiologically in both carotid arteries. Unfortunately, the residues of brain tissue were too small to provide a representative investigation of the CNS. Nevertheless, we have some indirect evidence for slightly reduced brain function of Count Heinrich during the last few years of his life. This is seen by his gradually more and more shaky hand writing (see S6 File). As an example, the signature of Count Heinrich, which was very consistent until around his 75^th^ year, becomes somewhat “irregular” prior to his death. Although this represents only minor and indirect evidence, the combination of progressively shaky hand writing and significant arteriosclerosis of the carotid arteries supports the notion that he was at risk of developing neurological symptoms by chronic reduction in brain perfusion. Also the histological investigation of the utmost vascular periphery in small subcutaneous/ soft tissue arteries and arterioles did not reveal any evidence for microvascular arteriosclerosis. Since Count Heinrich was actively involved in the administration of the local Munich protestant church community until his death [15], this suggests that it is unlikely that he developed dementia.

Over the last few years there has been great debate about the presence and extent of arteriosclerosis in historic individuals. There is increasing evidence from various populations from all over the world and of different times that arteriosclerosis was a common feature [35–40]. The present case of arteriosclerosis contributes to this debate insofar as Count Heinrich suffered from significant arteriosclerosis, not very surprising given his advanced age. This in turn might have been the result of his high nutritional level. The similarly high physical activity level late in his life obviously did not stop arteriosclerosis. In this regard, a recent debate on the influence of chronic infectious diseases on the promotion of arteriosclerosis may be of interest [41] and warrants further data.

### Evidence for tuberculosis

This case presents evidence for long-standing pulmonary and extrapulmonary tuberculosis. As well as the cardiovascular disease we detected several calcifications in the lung parenchyma bilaterally with a prominent focus at the right pulmonary hilum. Histopathology revealed scar-like fibrosis with significant anthracosis in this focus, but no evidence for mycobacteria by both bacterioscopy and molecular investigation. Small calcified nodules at the periphery of both lungs were also free of evidence of active tuberculosis on histology, as well molecular tests for aDNA of the *M*. *tuberculosis* complex in several samples from both lungs, particularly in the lower lobes.

Additionally, the CT scans revealed an irregular defect of the second lumbar vertebral body with irregular and slightly sclerotic margins, consistent with tuberculous osteomyelitis. However, the analysis of small samples of bone adjacent to this osseous defect zone were negative in molecular tests for mycobacterial infection. This may possibly be due to the fact that we could not remove the complete lumbar vertebral body (in order to maintain the stability of the corpse after our autopsy when laying-out of the mummy during reburial. Similarly, previous studies had shown that a positive molecular test for *M*. *tuberculosis* complex aDNA is positive in around 80% of typical osseous vertebral lesions [21], but fails to detect the mycobacteria in around 20% of macroscopically suspicious cases.

Beyond the presumed old-healed pulmonary tuberculosis and the tuberculous vertebral osteomyelitis, we found on the CT scans an encapsulated, centrally cystic, tumour-like mass at the right retroperitoneum. This was located adjacent to the second lumbar defect and contained some streak-like calcifications. While this mass remained pathogenetically unclear during macroscopic analysis, histology (and even more the application of the special stain Ziehl-Neelsen; see also [41]) detected the presence of acid-resistant small bacilli in the cyst wall strongly suggesting an active tuberculous cold abscess along the psoas muscle. This was further confirmed by the molecular identification of a specific amplification product for *M*. *tuberculosis* complex aDNA and its further classification by spoligotyping as infection by a strain of the human variant of *M*. *tuberculosis senso stricto* [7,42].

This case was investigated by molecular techniques that may no longer be used for such an analysis. Nowadays, molecular whole-genome approaches, with next generation sequencing techniques, might have given more information on the molecular structure of the *M*. *tuberculosis* strain in Count Heinrich´s cold abscess. This was not available when this study started. Furthermore, the techniques applied here provide us with enough consistent information to draw clear conclusions, the molecular proof of an active *M*. *tuberculosis* infection, caused by a strain of the human *M*. *tuberculosis* type. In this regard, the lack of NGS does not change the amount of information.

The combination of these data strongly supports the notion that Count Heinrich LII. Reuss-Köstritz had suffered from primary pulmonary tuberculosis with (haematogenous) spread into the second lumbar vertebral body and from there to a cold abscess of the psoas region. The complete scarring of the primary complex in the lung, and the lack of detectable mycobacterial aDNA in that organ, suggests a long-standing time frame. This is further supported by the scar-like pseudocapsule of the cold abscess with focal streaky calcifications.

Encapsulated tuberculous abscesses of the retroperitoneum occur in only about 1–2% of cases with pulmonary tuberculosis. Accordingly, one quarter of active extrapulmonary tuberculosis affects the retroperitoneum [43]. The abscess mostly originates from lumbar skeletal tuberculous lesions (66,7%, [43]) such as suspected in this case. The primarily osseous inflammation follows the adjacent retroperitoneal fascial structures that envelop the psoas muscles, either directly or by lymphatic spread.

In present day populations, iliopsoas abscesses are rare, and mycobacterial infections comprise only a small number of those abscesses. Navarro Lopez et al. [44] reported tuberculous origin in only 12.1% of psoas abscesses. Others described similar rates (e.g. in a Korean study by Kim et al. [45] 14.7%). The outcome of the affected patient mostly depends on the pathogenetic development of the primary (lung) tuberculosis. This may have been different in historic populations where tuberculosis incidences were much higher [46,47] and the potential spread of the mycobacteria through the body was not influenced by modern intervention, such as antibiotic therapy. It has been estimated that about 5% of cases of spinal Pott´s disease (spinal tuberculosis) develop a psoas abscess [41]. The cold abscess itself, at least in the time period before antibacterial chemotherapy, frequently existed for a considerable period with good health and only limited clinical symptoms, such as flank or back pain and/or a slight fever. However, the chronic untreated cases had a mortality rate of 90% [39], particularly in immunocompromised individuals. In this regard, Count Heinrich’s case is unusual and suggests his optimal immune status due to the lack of major concomitant diseases, remaining physically and mentally active, and excellent nutrition.

The major risk in cold iliopsoas abscesses is dissemination of mycobacteria leading to systemic, (septicaemic) tuberculosis; in its most enhanced form termed “Landouzy sepsis”. Further risks from iliopsoas abscesses are bleeding and bacterial superinfection (which may also lead to systemic sepsis) [48,49]. In Count Heinrich LII. Reuss-Köstritz’s case, the abscess did not progress. At least from the extensive histomorphological and molecular analysis, the lung tissue, as the main target of septicaemic spread, remained free of evidence for *M*. *tuberculosis* infection.

Finally, this is not the first case of long-term survival in human *M*. *tuberculosis* infection that has been investigated molecularly. While this question is essentially unanswerable in those cases where only skeletal remains are present (the vast majority of cases)–in the more common smaller group of cases with sufficiently well preserved soft tissue (i.e. mummies), very few cases have been described. Likewise, the almost systemic analysis of well-preserved mummies from the Dominican crypt of Vac, Hungary, revealed cases with chronic tuberculosis with molecular positive mycobacterial aDNA [14]. However, these results were found in cases with latent tuberculosis, restricted to small pulmonary lesions. Interestingly, the oldest TB-positive case from Vac was a well-nourished 95-year-old woman [14].

Taking these observations together, we can conclude that chronically active tuberculosis in well-nourished and optimally supplied historic individuals may occur.

### Further pulmonary pathology

Beyond the obvious residues of healed pulmonary tuberculosis (with scar formation and calcifications) extensive histological analysis of lung tissue from both lungs and various lobes revealed significant differences between various regions. While parts of the left lower lobe showed a proteinaceous intra-alveolar exudate, samples from other lobes did not contain this feature. This focal intra-alveolar exudate is strongly suggestive of a focal inflammatory reaction. This rules out an exudation of blood plasma during the terminal systemic and/ or cardiac circulatory failure, as well as the complete inflammatory involvement of both lungs in a septicaemic process.

In summary, the histological analysis of lung tissue indicates a local pneumonic inflammatory process in line with the indicated cause of death in Count Heinrich´s death certificate. This indicates a “Lungenlähmung” (i.e. pulmonary palsy) as cause of death [15], a term that describes terminal pulmonary failure.

### Reconstruction of health aspects in Count Heinrich LII. Reuss-Köstritz and its implication for palaeopathology

The multidisciplinary investigation of the human remains of Count Heinrich identified several, potentially life-threatening diseases (persistent extrapulmonary tuberculosis, coronary and carotid arteriosclerosis, focal bronchopneumonia), and also allowed ranking of those diseases within the life history of the General. Likewise, we have good evidence that bronchopneumonia was his final cause of death. Coronary and brain vessel arteriosclerosis had occurred for some longer period before death (the brain vessel arteriosclerosis, presumably, for several years). The age of the lung and vertebral tuberculosis remained unclear, but the complete scarring of the right hilar lung lesions also favoured a long-term healing of, at least, several years. Similarly, the scarring and calcification of the cold iliopsoas abscess suggested a long-standing event.

Although we have no medical records for Count Heinrich´s life, we know that he had suffered from a very serious disease in the winter 1822/23, i.e. 28 years prior to his death [15]. Although very speculative, it is conceivable that this episode may have represented the (possibly initial) tuberculous infection that subsequently led to spread of the disease. In this case, a two decades plus old and active tuberculosis did not cause his death. This means that the presence of chronic infectious diseases such as tuberculosis, when found in a mummy, cannot be assumed to be the cause of death. We strongly advise caution in using this interpretation.

### The reasons for Count Heinrich’s final burial site

Ultimately, our study unravelled a mystery that we faced at the beginning, why had Count Heinrich LII. Reuss-Köstritz been buried in a Bavarian crypt, far away from his small homeland in Thuringia? All initial suspicions of a family association were ruled out using the biomolecular aDNA typing of Count Heinrich and the other inhabitants of the crypt (cf. S5 File, also [50]). Our investigation proved that Count Heinrich was not related to the Jordan family. The relationship between both former Royal Bavarian Generals was only on the basis of common war experience and friendship (for details suggesting this life-long friendship please refer to the count´s biography [15]). Finally, since the death of his second eldest brother Heinrich XLIX. in 1840 (see S1 File), the family crypt of the Counts of Reuss-Köstritz in Thuringia was entirely full so there was no space for Count Heinrich LII’s burial. This was the reason why we were able to investigate this historic individual in the vicinity of Munich.

## Supporting information

S1 File Time table of events during the life von Count Heinrich LII. Reuss-Köstritz. (PDF)

S2 File Juridical, ethical and practical considerations for the full scientific investigation of complete mummified human bodies. (PDF)

S3 File Molecular analyses—Analytical procedure and reagents and technical equipment used. (PDF)

S4 File Results of the contact radiography of internal organs and bone. (PDF)

S5 File Genotyping of mummy samples from all individuals of the Jordan crypt. (PDF)

S6 File Signatures of Count Heinrich LII. Reuss-Köstritz between 1833–1850. (JPG)

S1 Raw images (PDF)
