# Supplementary material for: Chronic active non-lethal human-type tuberculosis in a high royal Bavarian officer of Napoleonic times–a mummy study
Source: PLoS One. 2021 May 4;16(5):e0249955. doi: 10.1371/journal.pone.0249955 (PMC8096010; doi:10.1371/journal.pone.0249955)
Supplement: S1 File — (PDF) [file pone.0249955.s001.pdf]

Supplement S1

Time table of events during the life von Count Heinrich LII. Reuss-Köstritz

1763 09-21	Birth of Count Heinrich LII. the third son of Count Heinrich XXIII. and his wife Countess Maria Eleonora, neé Schönburg-Wechselburg in Köstritz
1768	Count Heinrich LII. suffers from whooping cough (*pertussis*)
1780 06-30	Entry into the Palatino-Bavarian army as a Sous-Lieutenant
1784	Count Heinrich LII. participates in a large military manoeuvre of the Palatino-Bavarian army
1784 08-06	Advancement to captain
1800 01-25	Nominated Commander of the First Grenadier Battalion and beginning of the War of the Second Coalition between France and Bavaria with Austria and England
1800 06-01	Reuss participates in the combat at Weissenhorn/Bavaria
1800 06-27	Participation in the combat at Neuburg/Donau/Bavaria
1800 10-03	First petition for release from the Palatino-Bavarian army declined by the Palatino-Bavarian elector Max Joseph
1800 12-03	Reuss participates in the Battle of Hohenlinden
1801 summer	Travels to Paris
1802 08-12	Commander of the Reconnaissance Corps against Austria
1803 06-07	Second petition for release from the Palatino-Bavarian army, accepted by the elector
1804 02-11	Re-engagement as General Major and Adjutant
1805 06-11	General Reuss appointed supervisor of the Bavarian Electoral Prince Ludwig on his trip to Italy
1805 11-05	Return of Prince Ludwig with General Reuss; meanwhile War of the Third Coalition with Franco-Bavaria against the Austro-Russian army
1806 01-26	Bavaria is kingdom from 1st January, 1806; Crown Prince Ludwig starts trip with general Reuss to France
1807 01-02	Prince Ludwig and General Reuss take over the commando of the Bavarian army corps during the War of the Fourth coalition, Franco-Bavaria against Prusso-Russia
1807 05-17	Participation in the battle of Pultusk/ Poland
1807 11-30	General Reuss accompanies the Bavarian Royal family to Italy
1808 10-15	General Reuss is with the Bavarian King Max Joseph at the “Three-Emperor-Meeting” at Erfurt/ Thuringia (Tsar Alexander, Emperor Joseph, and Napoleon)

1809 04-11	General Reuss accompanies the Royal family from Munich to Dillingen during the War of the Fifth coalition (Franco-Bavaria against Austria)
1809 12-11	Reuss joins the Royal family on their journey to Paris
1814 09-25	Reuss is part of the Bavarian Royal delegation to the Congress of Vienna
1816 04-14	Third petition of release from the Royal Bavarian army
1818 03-17	Reactivation as General Adjutant of the Royal Bavarian army
1819 01-23	General Reuss sent as emissary to the Royal Saxonian couple on behalf of their 50th matrimonial celebration
1821 05-27	Count Reuss appointed member of the managing committee of the Munich branch of the Bavarian protestant church
1822 11-09	Count Reuss transfers the (procura) wedding ring of the princely wedding between the Bavarian princess Amalia and the Saxonia prince Johann from Munich to Dresden
1822 11-21	Count Reuss stays with his sister in Waldenburg/ Saxonia for serious health reasons
1822 12-31	King Max Joseph wishes Count Reuss speedy recovery from his disease (not specified)
1823 04-11	Reuss back in service, accompanies the Bavarian King to Leipzig and Weimar
1823 09-21	Member of the first synod of the Bavarian protestant church
1823 11-21	Reuss transfers the (procura) wedding ring of the princely wedding between the Bavarian princess Elisabeth and the Prussian crown prince Friedrich Wilhelm IV
1824 10-30	Journey of the Royal Bavarian court including Reuss to Vienna on behalf of the wedding of the Bavarian princess Sophie with Prince Ferdinand
1825 11-18	After the death of King Max I. Joseph, fourth petition for release from the Royal Bavarian army
Since 1826	Regularly participates in various committees of the protestant church, the Royal court, and repeated excursions with members of the Royal family
1829 09-14	Death of Count Reuss´ sister in Waldenburg/Saxonia; he takes part in the funerary ceremonies
1833 03-07	Death of his eldest brother Count Reuss XLVII. in Waldenburg/ Saxonia; shortly after he journeys to Saxonia
1840 02-29	Death of his second eldest brother Count Reuss XLIX. in Köstritz/ Thuringia; Count Heinrich LII. takes over the duties as the leader of the Köstritz branch of Reuss, younger line; Count Heinrich, however, stays in Munich
1842	Count Heinrich LII. changes his apartment within Munich
Until 1850	Leading member of the Munich protestant Bible club participating regularly
1851 02-23	Death of Count Heinrich LII. in Munich from “respiratory paralysis”
