# Supplementary material for: Chronic active non-lethal human-type tuberculosis in a high royal Bavarian officer of Napoleonic times–a mummy study
Source: PLoS One. 2021 May 4;16(5):e0249955. doi: 10.1371/journal.pone.0249955 (PMC8096010; doi:10.1371/journal.pone.0249955)
Supplement: S2 File — (PDF) [file pone.0249955.s002.pdf]

Supplement S2

Juridical, ethical and practical considerations for the full scientific investigation of complete mummified human bodies

The scientific investigation of human mummies touches various aspects that must have to be considered prior to starting with the analysis. These include juridical aspects which may, however, differ between various countries. Furthermore, several ethical considerations have to be taken into account. And finally, the practical performance may require special considerations.

1.Juridical aspects (applies only within the Federal Republic of Germany).

Since this paper describes a human mummy present in Germany, only German law apply here (a detailed juridical evaluation of crypt burials is given in [1]). While human corpses lose their own legal capacity at death, legal regulations exclude that a human corpse is regarded as a "matter" that can be owned by another subject ("*res extra commercium*") [1]. In consequence, usually the living next-of-kin, or if there is none, the state – i.e. the executor of the death care, has to organise a dignified funeral. Furthermore, these usually are also the executors of the last will. The only exclusions are impoundment by public prosecution services (for the clarification of the circumstances of an unlawful, and any subsequent legal procedures) or pre-mortem donation of the corpse consented whilst living to (non-profit) scientific institutions, such as anatomical teaching institutes.

This condition changes after a considerable period of time when the aforementioned executor of the death care is no longer living. The existing bodily remains, either skeletal or mummified corpus, gain the status of "matter" which may then be juridically the property of the cemetery owner. In this respect in Germany, a minimum waiting period of 110 years from birth or 30 years after death has to be observed (which is in parallel to German data protection regulations), before such a status may apply, provided that the aforementioned executor is no longer alive, or agrees. In these circumstances, the remains can be handled as matter, on the further condition that certain ethical considerations are observed (see below).

In Germany, most cemeteries are operated by the local community authorities and are therefore in public possession. However, particularly older cemeteries, often associated with a church, they may still be in the hands of the Church. In both instances, opening of the tombs and the eventual investigation of human remains are dependent on the consent of the owner [1].

Skeletal remains detected during construction work usually are handed over to the responsible state authorities, such as the state collections for anthropology. In these circumstances, the authority gains ownership; this holds also true for museums and other collections [2,3].

In the case of the crypt of Dötting/ Wackerstein, Baron Wilhelm von Jordan had erected the building in 1836 on his own ground and had handed over the tiny piece of land to a church foundation that juridically still exists and that is administrated by the Catholic Church, represented by the Diocese of Regensburg.

2. Ethical aspects

The scientific investigation of human mummified remains is a centre piece of human anthropology and paleopathology. Since both disciplines are dedicated to the elucidation of important knowledge on human history, there is no doubt about their scientific value and their position in science. This, however, requires respectful ethical handling of the human remains that should balance scientific curiosity with the optimal way to preserve those remains. Furthermore, the ethically correct way of handling is often dependent on the local religious and ethical standards which may differ from country to country across the world.

In Central Europe the ethical standards are significantly influenced by the recommendations of the various Christian churches. These clearly permit scientific investigations, particularly when these contribute to the overall knowledge to the benefit of mankind.

With respect to skeletal remains, it seems very easy to find the aforementioned balance, since the removal of isolated bones or bone parts for scientifically important analyses is not usually a cause for discussion. More problematic is the handling of mummified remains. While fragments, or otherwise naturally damaged mummies or parts of mummies, mostly provide easy access to target tissues and organs that may have still survived, the investigation of complete mummies is much more problematic. It is clear that in those instances the extent of any further damage should be kept to a minimum. However, it is also without doubt that even the most modern non-invasive analytical techniques typically based on x-rays, do some harm to the tissue and the long-term effects of such extensive radiation is still unknown. Furthermore, despite all technical progress in paleoradiology, it still cannot resolve all uncertain findings, such as in the present study where both the unclear cystic structure and the irregular shell-like objects in thorax and abdomen could not be further evaluated, despite the radiological studies being performed independently by two very experienced radiologists (one expert in paleoradiology). A previous study from a Korean mummy project highlights the limitations of CT-scans, using a simultaneous analysis of CTs and autopsy findings [4].

In 2003, the doyen of paleopathology, the American pathologist Arthur C. Aufderheide [5] stated about anatomical dissection methods; “The study of morphological changes in the tissue of mummified human remains is still the cornerstone upon which the science of mummy studies is based.” Although techniques have improved, this statement holds still true today.

The second important issue is, that even small-sized openings for minimally-invasive techniques by endoscopy penetrate the body wall, and these openings mostly cannot be closed. Additionally, the minimally- invasive approach is excellent if a target is already identified, but helps little when the underlying process is unknown or uncertain. Without doubt drilling a dozen holes into a mummy harms the corpse more significantly than one opening that can be closed properly at the end of the investigation.

In the present case, a minimally-invasive technique would clearly have missed the final correct cause of death, since the pneumonic insudations would not have been the target of a CT-guided approach.

3. Practical aspects

Finally, taking the aforementioned juridical and ethical considerations into account, a few practical aspects should be discussed, particularly with respect to Count Heinrich LII. Reuß-Köstritz’s mummy.

Beyond the ethical aspects that a mummy should not be mutilated by the investigations, we performed a modification of present-day autopsy technique by using a dorsal approach. This allowed a complete conservation of the ventral thorax and abdominal wall allowed appropriate presentation of the corpse for the return ceremony in 2012 and was easily accomplished. The dorsal opening strategy has the major advantage that it provides access to the lungs, the heart and the residues of abdominal organs. The obtained samples were sufficient enough for all subsequent investigations.

To get access to the dorsal body wall, the overlying clothing had to be removed and was easily achieved by unpicking the stitches of the seam of the clothing along the vertebral line. These were then re-stitched at the end of the examination and the clothing arranged appropriately. This approach also parallels the usual present-day practice for clothing of human corpses where the dorsal part of the clothing is cut and re-stitched after it has been put back onto the cadaver.

A further advantage of the dorsal approach is that the windows can be easily and firmly closed keeping the dorsal body wall intact; this is not possible with multiple endoscopic portals in the body wall. Particular aspects of the dorsal access approach are shown in the enclosed figures A and B.

4. References for this supplement:

1. Hönings L (2014) Rechtswissenschaftliche Grundlage im Umgang mit (historischen) Gruftbestattungen. In: Preuß D et al. (eds.) Grüfte retten! Ein Leitfaden zum pietätvollen Umgang mit historischen Grüften. Fachhochschulverlag, Frankfurt a. M., pp. 23-34.

2. Preuß D (2007) ... et in pulverem reverteris? Vom ethisch verantwortetem Umgang mit menschlichen Überresten in Sammlungen sowie musealen und sakralen Räumen, München.

3. Radekamp V (2013) Empfehlungen zum Umgang mit menschlichen Überresten in Museen und Sammlungen«, (ed.) Deutscher Museumsbund e.V., Berlin, pp.1–69.

4. Lim DS, Lee IS, Choi KJ, Lee SD, Oh CS, Kim YS, Bok GD, Kim MJ, Yi YS, Lee EJ, Shin DH (2008) The potential for non-invasive study of mummies: validation of the use of computerized tomography by post factum dissection and histological examination of a 17th century female Korean mummy. J. Anat. 213: 482-495.

5. Aufderheide AC (2003) The scientific study of mummies. Cambridge University Press, Cambridge, pp. 322-334.

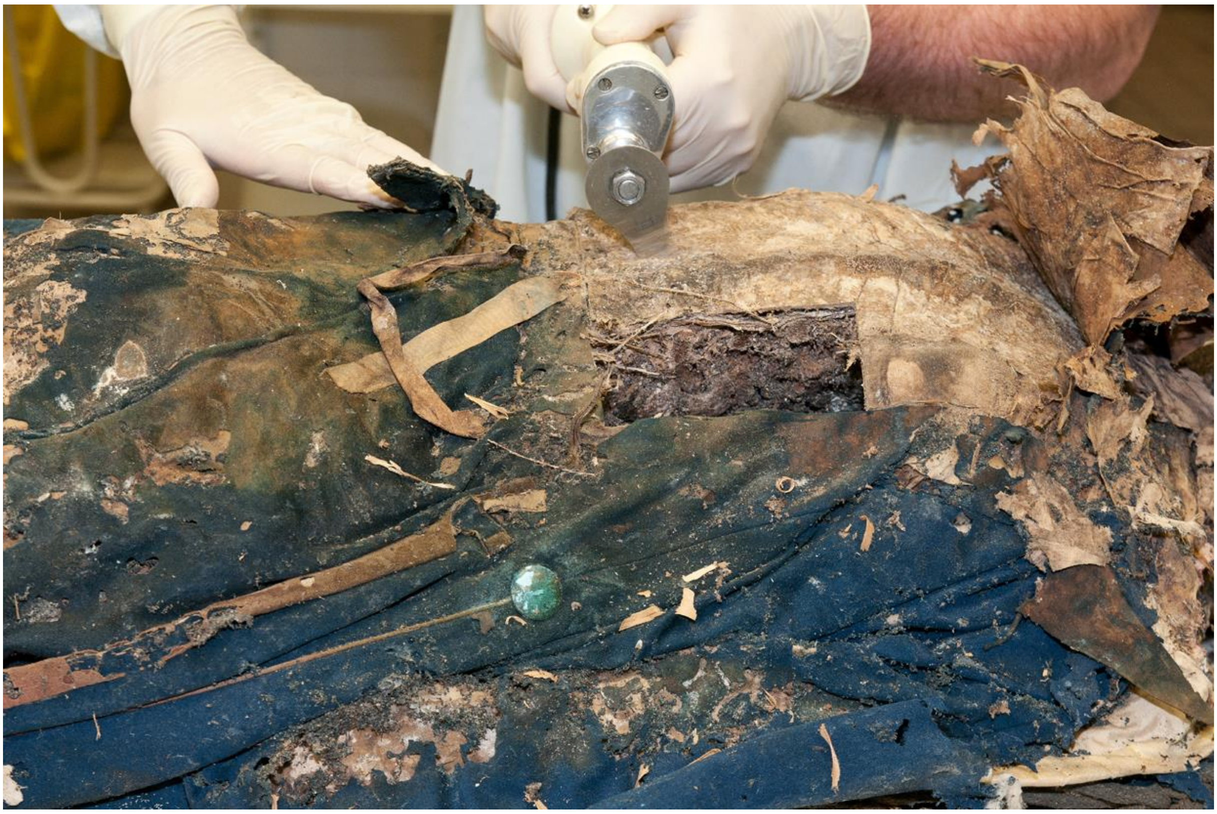

Fig. A – Autopsy procedure of the mummy. Following careful removal of the dorsal parts of the uniform, we opened the body with an oscillation saw.

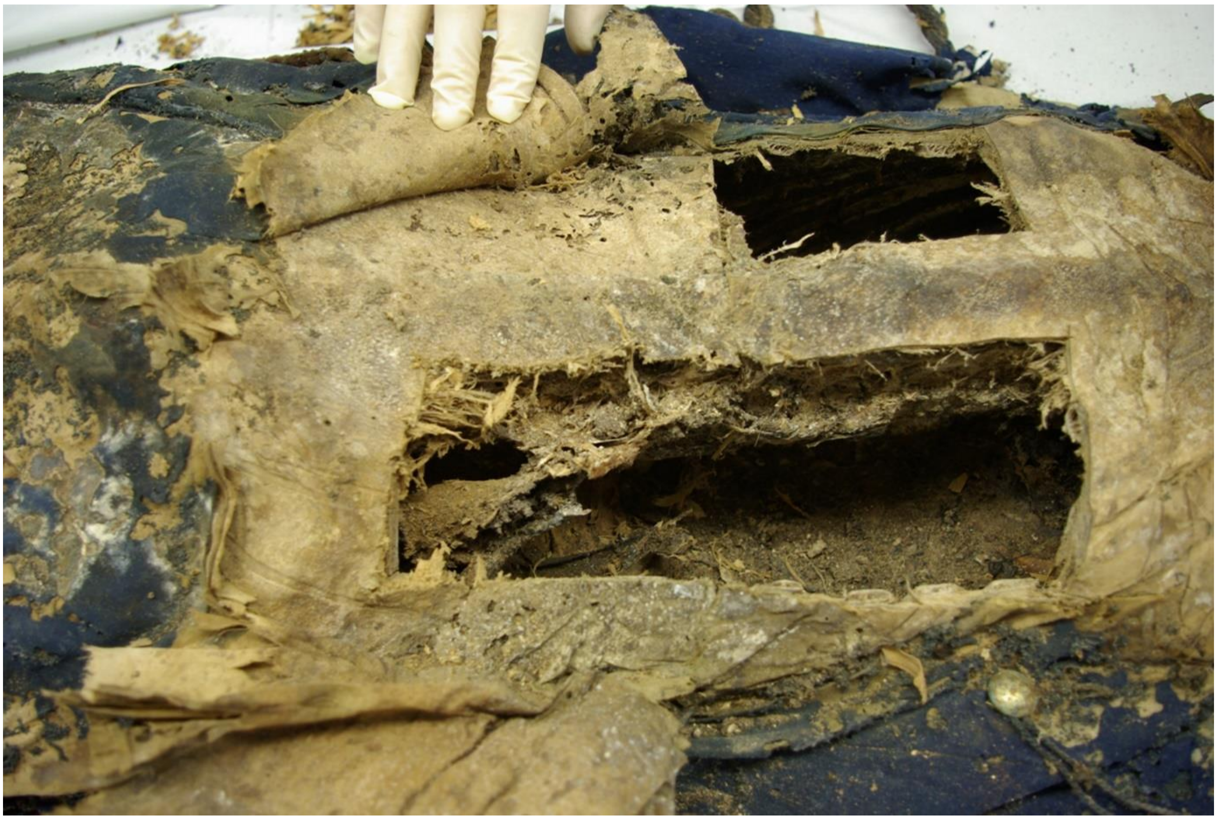

Fig B – The dorsal aspect of the mummy after cutting two windows. These show the enlarged access on the right side in order to investigate the right lower abdomen/ retroperitoneum.
