# Supplementary material for: Chronic active non-lethal human-type tuberculosis in a high royal Bavarian officer of Napoleonic times–a mummy study
Source: PLoS One. 2021 May 4;16(5):e0249955. doi: 10.1371/journal.pone.0249955 (PMC8096010; doi:10.1371/journal.pone.0249955)
Supplement: S3 File — (PDF) [file pone.0249955.s003.pdf]

Supplement S3

Molecular analyses – analytical procedure and reagents and technical equipment used

1.DNA extraction

Mummy organs and bone samples that had been obtained during mummy autopsy under contamination-free conditions as far as possible were photographed and x-rayed (contact x-rays). Tissue samples were then removed under sterile conditions of appropriate regions that had been identified on the x-rays (calcifications/ areas of reduced radiolucency) were minced mechanically and pulverized in a mixer mill (Retsch RM200, Haan, Germany) until complete pulverization. Ca. 500 mg of the bone material was then incubated with 2 mL of 0.5 M EDTA-solution containing proteinase K (0.25 mg/ml) at room temperature for 2 days on a rotary mixer as previously described (Haas et al., 2000). Organ material was subjected to a similar procedure for 1 day without EDTA in buffered saline. After centrifugation for 15 min. at 3,000 g, 0,5 mL of the supernatant was removed and 1 mL guanidine isothiocyanate solution with diatomaceous earth was added [1]. After incubation on a rotary mixer for another 2 h, the diatomaceous earth was pelleted by centrifugation and washed twice with 70% ethanol and one with acetone. The DNA was eluted with 80 µL sterile water. Finally, another washing and concentration step was performed with Microcon 30 filters (Millipore, Bedford, USA) and the final DNA solution was diluted to 20 µL with sterile water.

2.Amplification and analysis of human DNA

After determining the amount of human DNA per sample using realtime PCR Quantifiler ® Duo DNA Quantification Kit (Thermo Fischer Scientific) according to established protocols [2]. Individual STR-patterns were determined with the help of three different multiplex kits (Powerplex ® ESX17 fast system, Promega; AmpFISTR ® MiniFiler TM; AmpFISTR ® NGM Select TM PCR Amplification Kits, Thermo Fisher Scientific) in an AB Viriti thermal cycler according to the manufacturer´s instructions, using 32 and 34 cycle programs. The PCR products were analysed on an AB 3500xL capillary electrophoresis system (Thermo Fisher Scientific) and data analysis was carried out using the GeneMapper® ID-X Software v1.4 (Thermo Fisher Scientific). Consensus profiles were generated for each sample and compared between each other.

3.Amplification of mycobacterial DNA

For the specific amplification of mycobacterial DNA, a primer pair of a 123 base pair segment of the repetitive sequence IS6110 of M. tuberculosis was amplified as previously reported [3]. The PCR reaction mix contained 10 mM Tris-HCl (pH 8.3), 50 mM KCl, 1.5 mM MgCL2, 200 µM of each deoxynucleotide trisphosphate (Amersham Pharmacia, Uppsala, Sweden), 1 µM of each primer, 0.025 U/µL AmpliTaq Gold (PE Biosystems. Foster City, USA) and 0.5 µL extracted DNA to a final volume of 20 µL. PCR conditions were as follows: 10 min at 95°C followed by 45 cycles of 94°C for 1 min, 66°C for 1 min, and 72°C for 1 min. After the final cycle, another 8 min at 72°C was added [1].

3. Detection of PCR products of the M. tuberculosis complex.

The PCR products were electrophoresed on a 4% agarose gel and visualized in a UV scree after staining with ethidium bromide [1].

4. Sequence analysis of PCR products

For subsequent further analysis of the resulting amplicons, these were excised from the agarose gel. The resulting sample was then used to elute the DNA form the gel by a purification kit (Freeze´n Squeeze, Bio-Rad, Hercules, USA). With this eluted DNA a cycle sequencing was performed with a dye-terminator cycle sequencing kit (PE Biosystems). Automatic sequencing was performed on an ABI PRISM 310 Genetic Analyzer (PE Biosystems).

5. Spoligotyping

Spoligotyping was used for further analysis of the sample with a positive signal for the IS6110 region. The method was performed as described by Kamerbeek et al. [4], except that the cycle number was increased to 45 cycles. For this analysis, we used a commercial spoligotyping kit (Isogen, Maarsen, The Netherlands). The extracted DNA was amplified with primers DRa and DRb, which enable the amplification of the whole DR region, so that the spacers between the DR targets can be evaluated. A biotin-labelled reverse primer, Dra, was used to obtain biotin labelled PCR products after amplification. Subsequently, the PCR products were perpendicularly hybridized to lines of immobilized spacer oligonucleotides that represent spacers of known sequence. The oligonucleotides were covalently linked to an activated membrane in parallel lines. Following the hybridization, the membrane was incubated with streptavidin peroxidase. For the detection of the hybridization signals, an enhanced chemiluminescence (ECL) system was used (Amersham Pharmacia). In this system, peroxidase catalyzed a reaction, resulting in the emission of light, which can be visualized by autoradiography (Hyperfilm ECL; Amersham Pharmacia). The resulting spoligotyping patterns were compared to an international database that contained a total of 813 spoligotyping patterns reported at least twice, representative of 11,708 clinical isolates from more than 90 countries [5].

References for the supplement:

1.Haas CJ, Zink A, Molnar E, Szeimies U, Reischl U, Marcsik A, Ardagna Y, Dutour O, Plafi G, Nerlich AG (2000) Molecular evidence for different stages of tuberculosis in ancient bone samples from Hungary. Am. J. Phys. Anthropol. 113: 293-304.

2. Anslinger K, Bayer B (2011) The effect of increased cycle numbers using the Quantifiler ® Duo DNA Quantification Kit (AB) on the detection of minute amounts of male DNA in mixtures and its application in routine case work. Forens. Sci. Int. Genet. Suppl. Series 3: e277-278.

3. Eisenach KD, Cave MD, Bates JH, Crawford JT (1990) Polymerase chain reaction amplification of a repetitive DNA sequence specific for Mycobacterium tuberculosis. J. Infect. Dis. 161:977–981.

4. Kamerbeek J, Schouls L, Kolk A, Agterveld A, van Soolingen D, Kuijper S, Bunschoten A, Molhuizen H, Shaw R, Goyal M, van Embden JDA (1997) Simultaneous detection and strain differentiation of Mycobacterium tuberculosis for diagnosis and epidemiology. J. Clin. Microbiol. 35:907–914.

5. Zink AR, Sola C, Reischl U, Grabner W, Rastogi N, Wolf H, Nerlich AG (2003) Characterization of Mycobacterium tuberculosis complex DNAs from Egyptian mummies by spoligotyping. J. Clin. Microbiol. 41: 359-367.

Uncropped and unmounted gel/blot images:

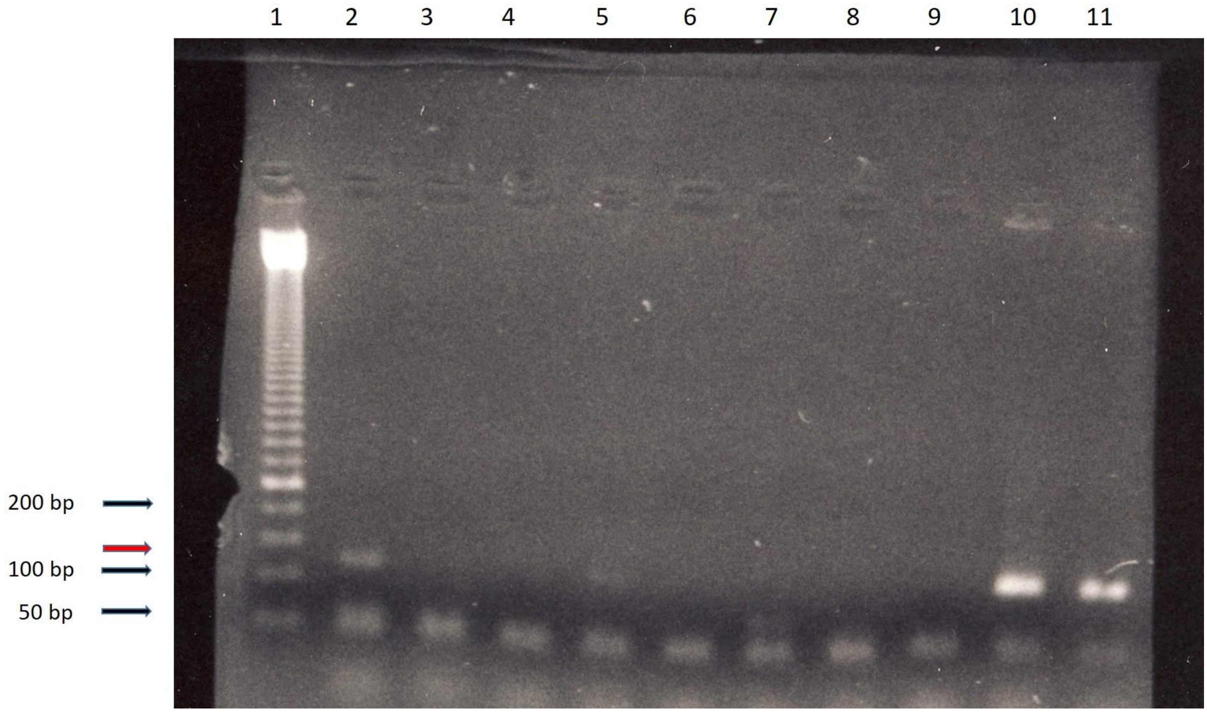

Figure 18: Gel electrophoresis for IS6110: lane 1 = standard; 2 = ”cyst wall”, 3-6: various lung samples; 7 right lung hilum; 8 vertebral body L2; 9 negative control; 10-11 positive controls (previously proven case of MTB from ancient Egypt). Red arrow: Target amplicon of 123 bp size.

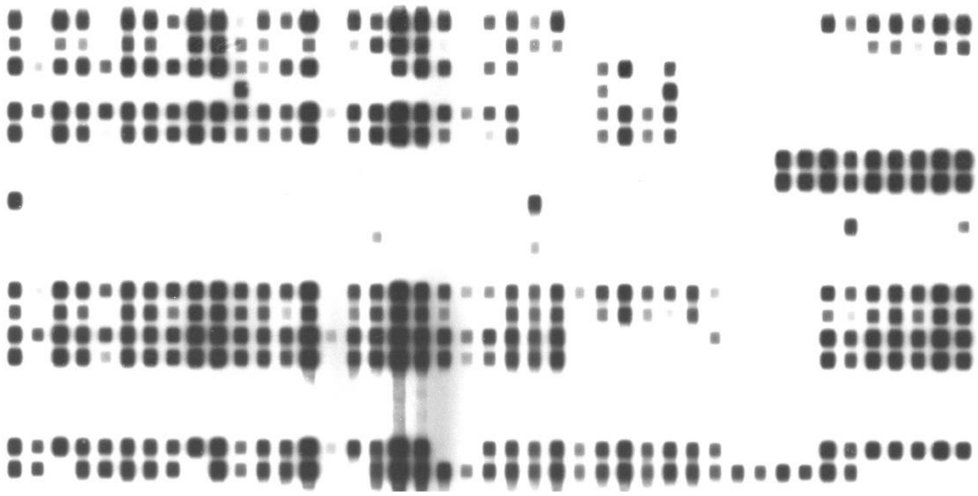

Figure 19: Original spoligotyping blot of various TB cases. General Reuß = lane 13; M. tb H37Rv control = lane 20; M. bov. BCGP3 control = lane 21.
