# Supplementary material for: Chronic active non-lethal human-type tuberculosis in a high royal Bavarian officer of Napoleonic times–a mummy study
Source: PLoS One. 2021 May 4;16(5):e0249955. doi: 10.1371/journal.pone.0249955 (PMC8096010; doi:10.1371/journal.pone.0249955)
Supplement: S4 File — (PDF) [file pone.0249955.s004.pdf]

Supplement S4

Results of the contact radiography of internal organs and bone.

All removed major tissue samples or organs underwent contact radiography in order to identify areas of different radiodensity and especially calcification. These were done by routine plain radiological investigation (70kV). Thereby, the exact removal of samples for subsequent histological and/or molecular investigations was possible. This approach confirmed the small calcifications in both lungs with a major calcification area in the right lung centrally, and several specks in the periphery of both lungs (Fig. A). The retroperitoneal cyst also contained flake-like calcifications most obvious in its caudal part, but was otherwise without major calcium deposits. (Fig. B). The heart was remarkable since all three functional coronary arteries revealed streaky calcifications along their course, but there were no calcifications within the obvious remnants of the heart muscle (Fig. C). Finally, the bone of the vertebral column showed dense bone tissue fairly unexpected for the individual´s old age. However, the bridging spondylarthrosis seen in the CT-scans of the vertebral column was well visible on the contact radiographs (Fig. D), consistent with Diffuse Idiopathic Skeletal Hyperostosis (DISH).

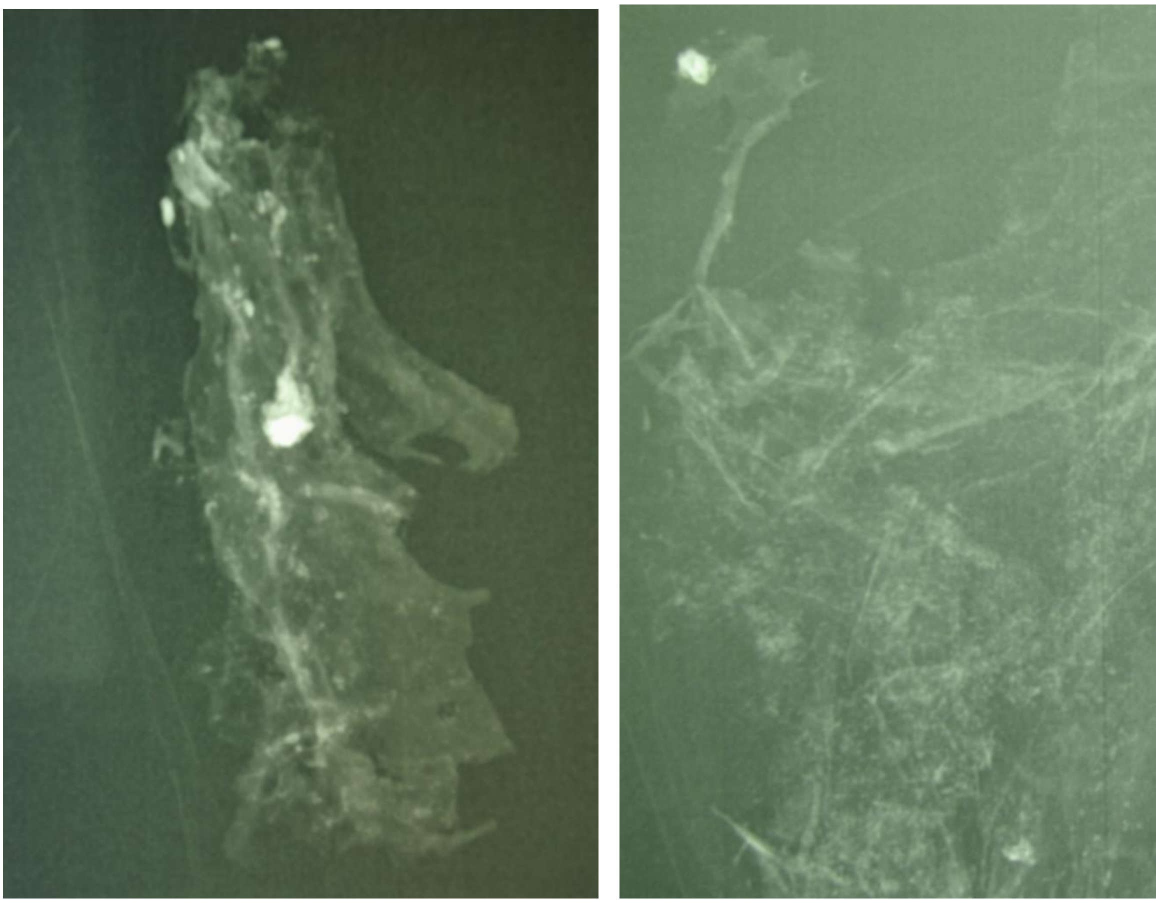

Fig A – Contact plain radiographs of both lungs (A: right; B: left) confirming the multiple small calcifications, particularly at the right hilum

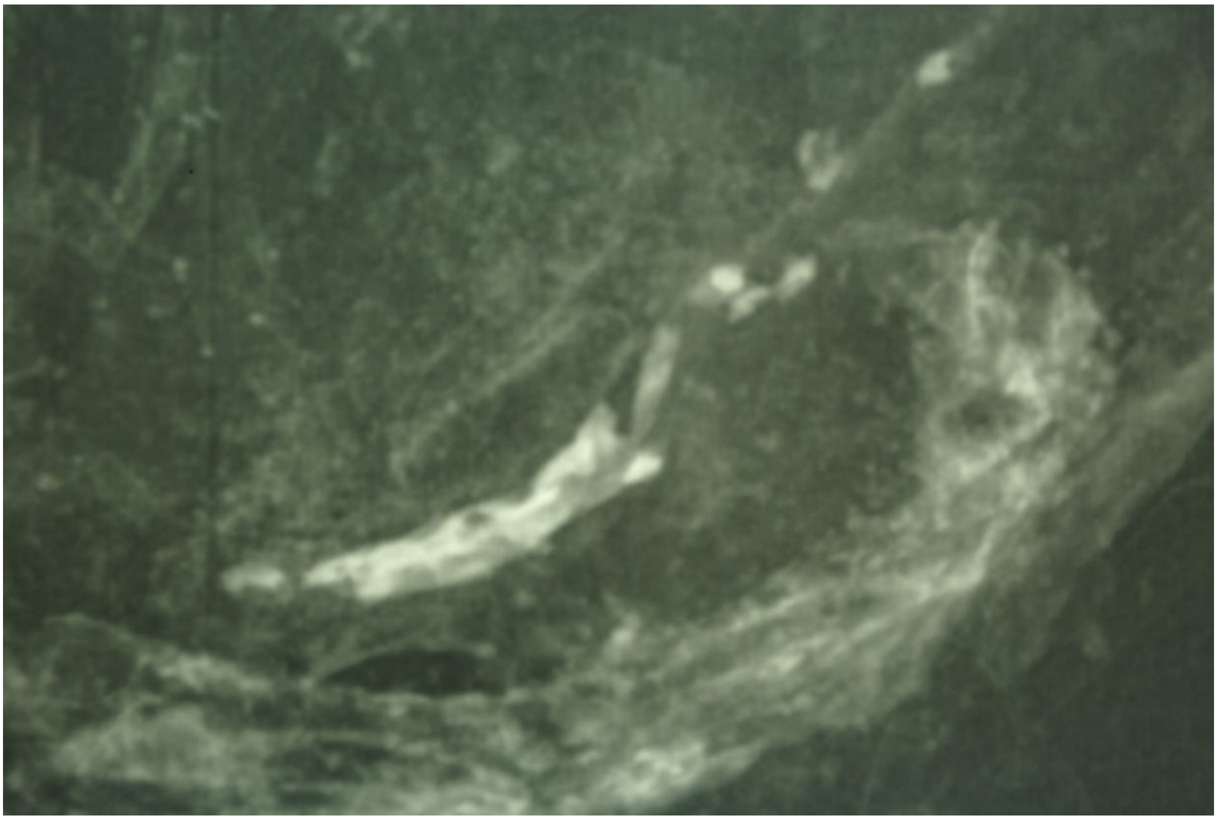

Fig B – Plain radiograph of the removed retroperitoneal cyst showing focal streaky and spot-like calcifications

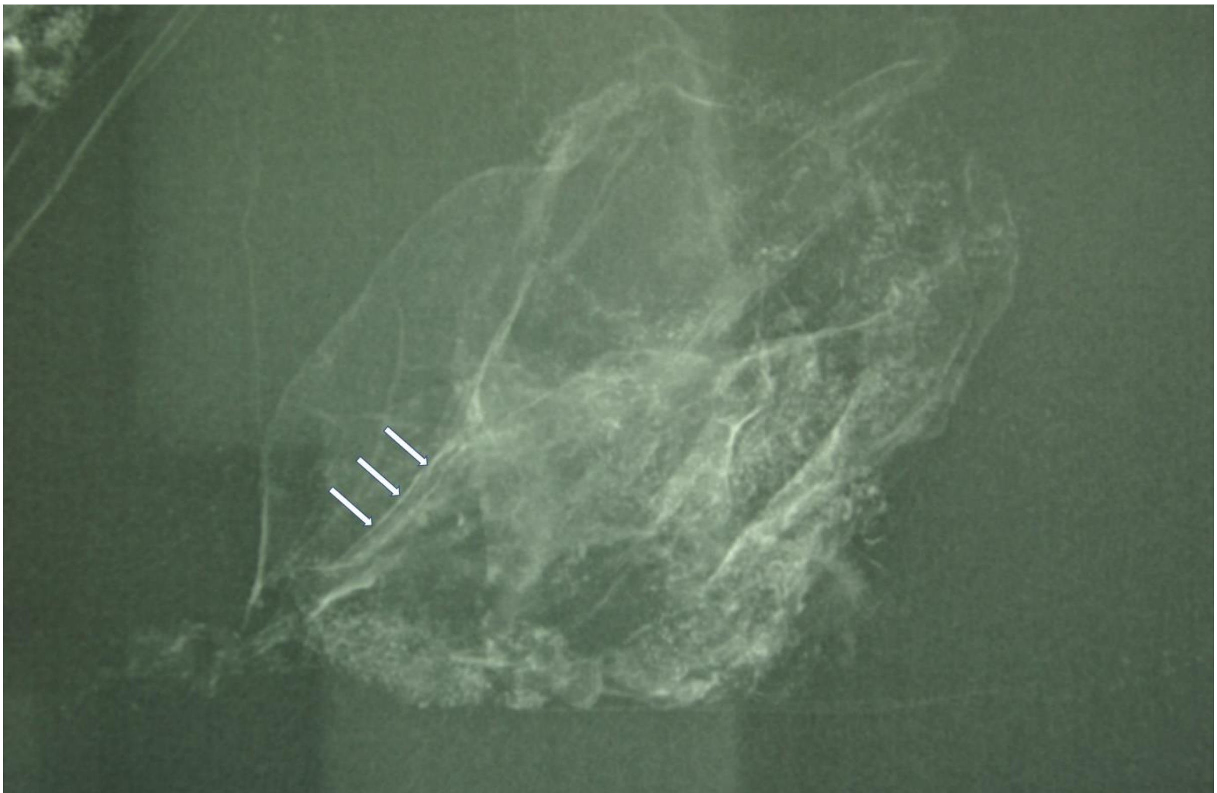

Fig C – Contact radiograph of the heart following autoptic removal. Note the “double-track” vascular calcifications of the coronary arteries (arrows).

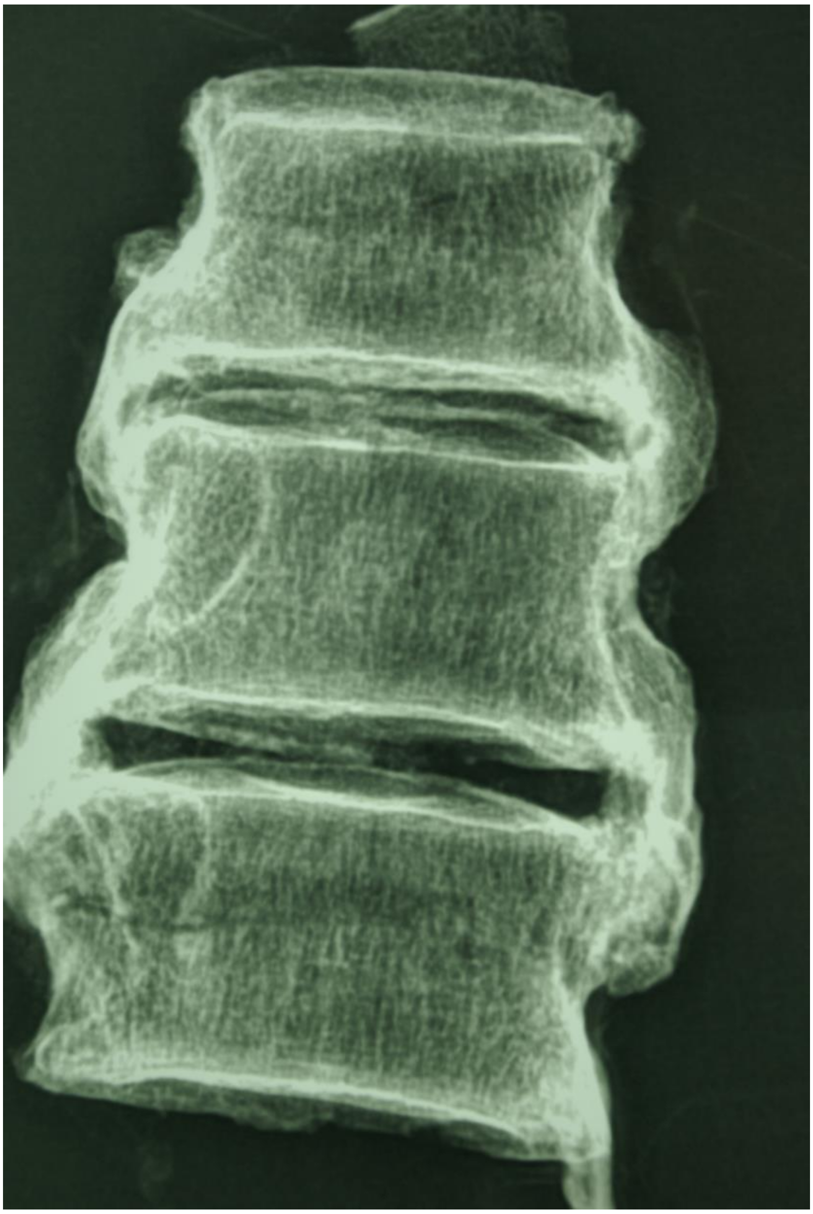

Fig D – Contact radiograph of three-level segment of the vertebral column confirming the bridging osteophytosis, a sign of DISH. Note also the otherwise excellent bone architecture.
