# Supplementary material for: Chronic active non-lethal human-type tuberculosis in a high royal Bavarian officer of Napoleonic times–a mummy study
Source: PLoS One. 2021 May 4;16(5):e0249955. doi: 10.1371/journal.pone.0249955 (PMC8096010; doi:10.1371/journal.pone.0249955)
Supplement: S5 File — (PDF) [file pone.0249955.s005.pdf]

**Supplement 5**

**Genotyping of mummy samples from all individuals of the Jordan crypt**, Wackerstein [15].

The STR-profile is presented for all 5 individuals of the crypt thereby excluding direct family relationships between **Count Heinrich LII. Reuss-Köstritz** and the Jordan family.

| *Individual* | *D3S1358* | *VWA* | *FIBRA* | *THO1* | *SE33* | *D8S1179* | *D21S11* | *D18S51* | *Amel* |
|---|---|---|---|---|---|---|---|---|---|
| Wilhelm | 15/16 | 14/19 | 22/22 | 6/9.3 | 20/28.2 | 8/13 | 28/30 | 14/17 | X/Y |
| Violande | 15/15 | 15/18 | 20/23 | 6/8 | 23/33.2 | 8/12 | 28/29 | 14/17 | X/X |
| Max | 15/15 | 14/18 | 22/23 | 6/8 | 28.2/33.2 | 12/13 | 28/28 | 17/17 | X/Y |
| Carolina | 15/15 | 15/19 | 22/23 | -- | -- | -- | -- | 17/17 | X/X |
| **Heinrich** | **14/18** | **18/19** | **21/23** | **--** | **29.2/n** | **10/15** | **31/34.2** | **13/(14)** | **X/Y** |

| *Individual* | *D16S539* | *D2S1338* | *D19S433* | *D22S1045* | *D1S1656* | *D10S1248* | *D2S441* | *D12S391* | |
|---|---|---|---|---|---|---|---|---|---|
| Wilhelm | 12/13 | 17/21 | 12/15 | 11/15 | 12/16 | 13/15 | 11/14 | 17/17 | |
| Violande | 11/11 | 17/25 | 14/15 | 16/16 | 11/16 | 14/15 | 12/14 | 15/19 | |
| Max | 11/12 | 17/17 | 15/15 | 15/16 | 12/16 | 15/15 | 11/14 | 17/19 | |
| Carolina | -- | 17/(25) | 12/15 | 15/16 | 12/n | 15/n | 14/n | 15/17 | |
| **Heinrich** | **9/11** | **19/20** | **13/14** | **15/(16)** | **12/15.3** | **13/16** | **11/11** | **18/20** | |

| *Individual* | *D13S317* | *D7S820* | *CFS1PO* | | | | | | |
|---|---|---|---|---|---|---|---|---|---|
| Wilhelm | 9/11 | 8/10 | 9/12 | | | | | | |
| Violande | 8/11 | 9/12 | 12/13 | | | | | | |
| Max | 11/11 | 9/10 | 12/12 | | | | | | |
| Carolina | 8/9 | -- | 12/13 | | | | | | |
| **Heinrich** | **12/13** | **11/13** | **10/11** | | | | | | |

n = second allele cannot be certainly determined

( ) = allele with lower intensity

Annotation: The individuals are: Wilhelm von Jordan (1775-1841 CE); Violande von Jordan, nee Countess Sandizell (1783-1859 CE); their son Max[imilian] von Jordan (1818-1850 CE);

their daughter Carolina von Jordan (1815-1816 CE, see also ref. [44]), Count Heinrich Reuss-Köstritz (1763-1851 CE);

The STR-profiles covered in total 20 markers. For Wilhelm, Violande and Max von Jordan the complete genotypes could be determined for all marker systems. The profile received for Heinrich Reuss-Köstritz consists of 18 markers showing a full genotype, 1 marker system where only one allele could be certainly detected, and one where no alleles could be detected at all. For Carolina von Jordan, too, only a partial, but nevertheless meaningful profile (consisting of 11 complete and 3 incomplete genotypes) could be obtained.

The comparison of the STR-Profile indicates the parentship of Wilhelm von Jordan and Violande von Jordan for their children to more than 99.99% probability; any closer genetic relationship between Count Heinrich Reuß-Köstritz and any member of the Jordan family is clearly excluded.
