# Supplementary figures and images for: Chronic active non-lethal human-type tuberculosis in a high royal Bavarian officer of Napoleonic times–a mummy study
Source: PLoS One. 2021 May 4;16(5):e0249955. doi: 10.1371/journal.pone.0249955 (PMC8096010; doi:10.1371/journal.pone.0249955)

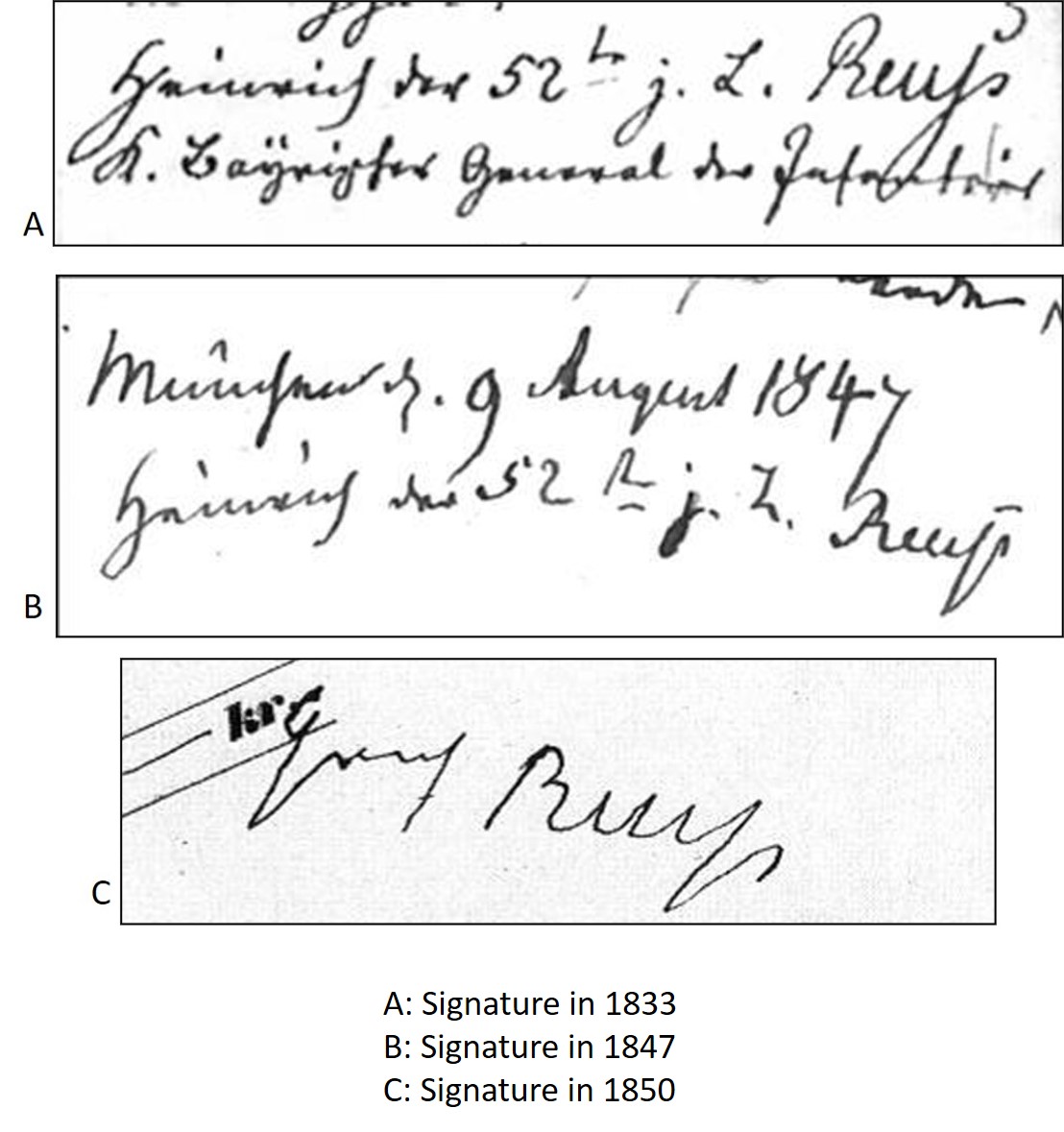

Supplement: S6 File — (JPG) [file pone.0249955.s006.jpg]

## Raw Images

Fig. 18:

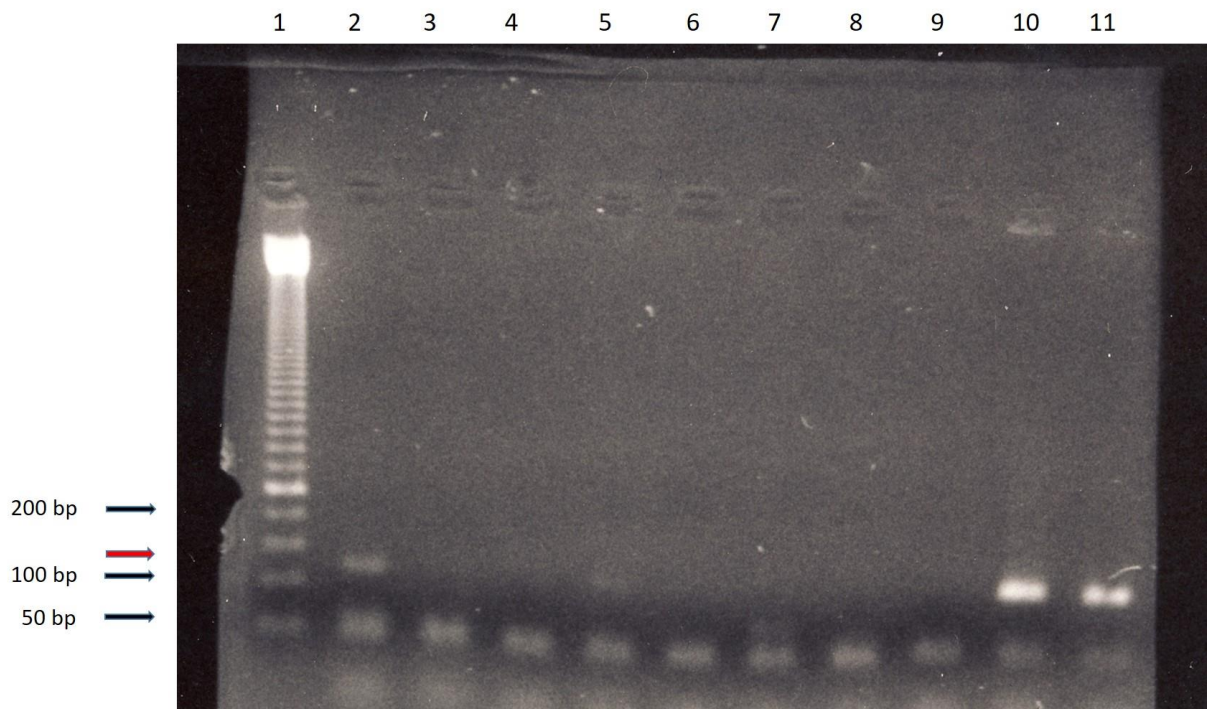

Fig. 19:

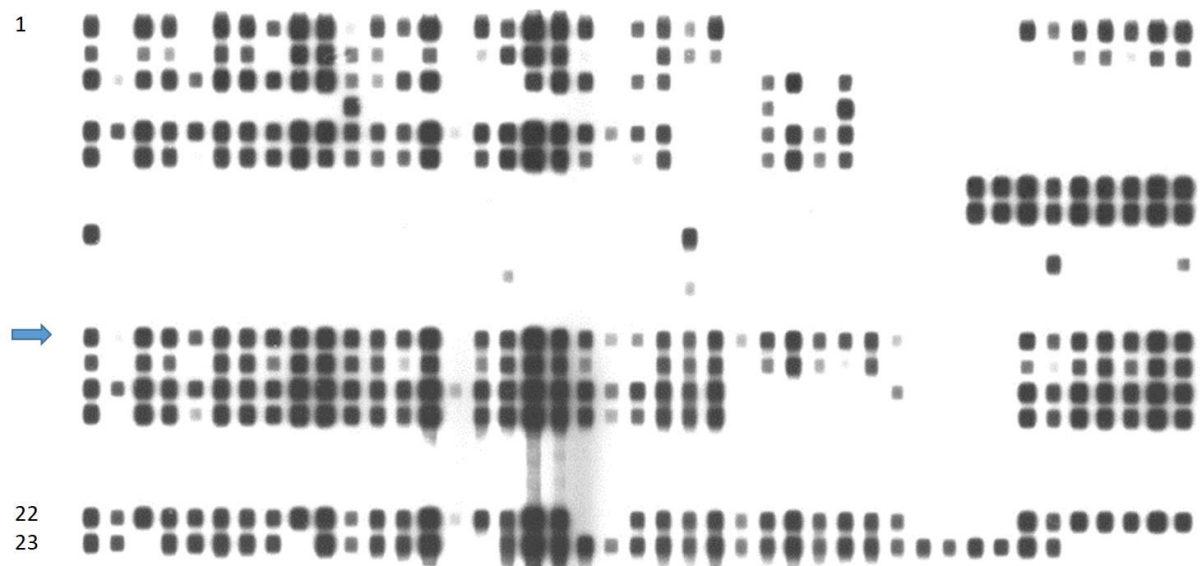

Supplement: S1 Raw images — (PDF) [file pone.0249955.s007.pdf]
